# Plasma Extracellular Vesicles Enriched for Neuronal Origin: A Potential Window into Brain Pathologic Processes

**DOI:** 10.3389/fnins.2017.00278

**Published:** 2017-05-22

**Authors:** Maja Mustapic, Erez Eitan, John K. Werner, Sean T. Berkowitz, Michael P. Lazaropoulos, Joyce Tran, Edward J. Goetzl, Dimitrios Kapogiannis

**Affiliations:** ^1^Intramural Research Program, Laboratory of Neurosciences, National Institute on Aging, National Institutes of Health (NIA/NIH)Baltimore, MD, United States; ^2^Department of Neurology, Johns Hopkins School of Medicine, Johns Hopkins UniversityBaltimore, MD, United States; ^3^Department of Medicine, University of California, San FranciscoSan Francisco, CA, United States; ^4^Jewish Home of San FranciscoSan Francisco, CA, United States

**Keywords:** extracellular vesicles (EVs), biological markers, Alzheimer's disease, phosphorylated tau protein, liquid biopsy diagnostics

## Abstract

Our team has been a pioneer in harvesting extracellular vesicles (EVs) enriched for neuronal origin from peripheral blood and using them as a biomarker discovery platform for neurological disorders. This methodology has demonstrated excellent diagnostic and predictive performance for Alzheimer's and other neurodegenerative diseases in multiple studies, providing a strong proof of concept for this approach. Here, we describe our methodology in detail and offer further evidence that isolated EVs are enriched for neuronal origin. In addition, we present evidence that EVs enriched for neuronal origin represent a more sensitive and accurate base for biomarkers than plasma, serum, or non-enriched total plasma EVs. Finally, we proceed to investigate the protein content of EVs enriched for neuronal origin and compare it with other relevant enriched and non-enriched populations of plasma EVs. Neuronal-origin enriched plasma EVs contain higher levels of signaling molecules of great interest for cellular metabolism, survival, and repair, which may be useful as biomarkers and to follow response to therapeutic interventions in a mechanism-specific manner.

## The need for neurodegenerative disease biomarkers

Neurodegenerative diseases, such as Alzheimer's and Parkinson disease (AD, PD), have an insidious course with a long preclinical phase. This lengthy prodromal period makes it difficult to identify patients earlier in the disease process when they might benefit the most from disease-modifying interventions. Given that these early pathological changes are asymptomatic, biomarkers rather than clinical manifestations are necessary for early identification and longitudinal follow up of future cases (Jack et al., 2010; Sperling et al., 2011; Kalia and Lang, 2016). Existing biofluid and imaging biomarkers for these diseases are imperfect, as their dynamic range does not span the entire course of the disease (Jack et al., 2010) and their classification accuracy falls below what is accepted for clinical diagnosis. These current limitations prevent promising biomarker research measures from being adopted for clinical practice (McKhann et al., 2011). In addition, MRI and PET-based biomarkers are expensive, whereas CSF-based biomarkers are invasive, limiting their appeal to both patients and physicians. Wide availability of a non-invasive, low-cost technology is required for biomarkers to be suitable for clinical practice, as well as to channel early affected patients or individuals at risk to clinical trials. Moreover, biomarkers linked to the pathogenic process can reduce costs for clinical trials in two ways. First, providing eligibility criteria that enrich the participant population for the presence of pathology can decrease the number of people required to demonstrate an effect. Second, providing surrogate outcomes can shorten trial duration, demonstrate target engagement, and allow for definitive testing of mechanisms involved. Therefore, reliable blood-based biomarkers are considered essential for therapeutic progress to be made in these diseases (Reiman et al., 2012).

A limitation of biomarker studies in peripheral blood has been the inability to link biomarker levels to brain pathology, as well as the relative uncertainty of their tissue of origin. Our team has pioneered a new approach in biomarker discovery for AD based on plasma extracellular vesicles (EVs) enriched for neural origin as a means of gaining direct access to brain pathogenic processes.

## Extracellular vesicles—a primer

The term EVs refers to nanoscale particles that are comprised of a lipid bilayer membrane and variable cargo of DNA, RNA, and proteins. EVs can be isolated from all biological fluids; their presence reflecting a balance between secretion and uptake by the various local cell types. Exosomes are a class of EVs, classically defined as small spherical EVs, 30–150 nm in size, originating from the endosomal/multivesicular body system (Bellingham et al., 2012). This rigid definition is being called into question with the advance of research, which has come to view EVs as a continuum in terms of size, biogenesis, and molecular constitution (Kowal et al., 2016). Research on EVs has seen an exponential increase in recent years, demonstrating an impressive variety of cargoes, including proteins (collated in the ExoCarta/EVpedia dataset, Simpson et al., 2012; Kim et al., 2013) and RNA species, many of which constitute potential biomarkers.

## Extracellular vesicles are crossing the blood-brain barrier

Accumulating evidence shows that EVs can cross the blood-brain barrier (BBB) from both directions. Bio-distribution analysis of fluorescent or luciferase labeled EVs has provided ample evidence of EVs entering the brain from the periphery. These studies show that 0.5–2% of EVs from various cancer cell lines accumulate in the brain after being injected into the circulation (Lai et al., 2014; Hoshino et al., 2015; Wiklander et al., 2015). In addition, peripheral EVs were shown to deliver siRNA, miRNA, and mRNA to central targets after passing through the BBB (Alvarez-Erviti et al., 2011; Bala et al., 2015). These observations establish the therapeutic potential of EVs as a drug delivery platform. There is also evidence for exosomes crossing the BBB from the brain into the circulation. For example, glioblastoma specific mRNA has been found in circulating EVs and was also suggested as a biomarker (Skog et al., 2008; Noerholm et al., 2012; Chen et al., 2013). Following inflammation of the striatum, EVs have been shown to cross the BBB and recruit neutrophils from the liver. In addition, astrocyte-derived EVs were also found in the circulation (Goetzl et al., 2016b, Dr. Norman J. Haughey personal communication). We present evidence (below) that brain EVs can be recovered from peripheral blood of genetically modified animals.

## Two-step isolation of a EV subpopulation enriched for neuronal origin

**Figure 1:**
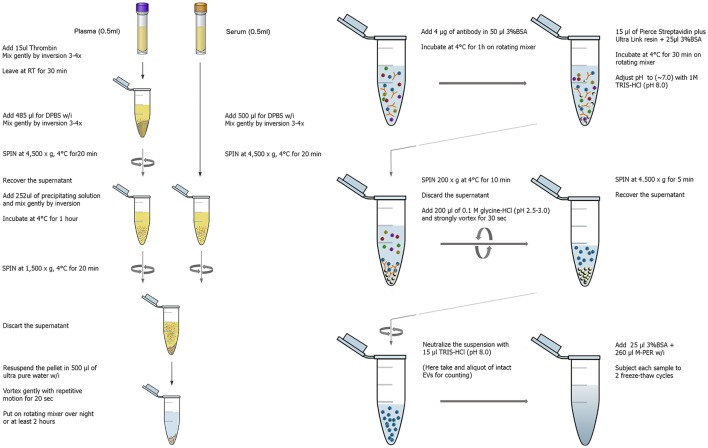
**Graphical workflow of the neuronal enrichment protocol**. W/i signifies the addition of protease and phosphatase inhibitors containing 2–3x the concentration recommended by the manufacturer.

**Figure 2:**
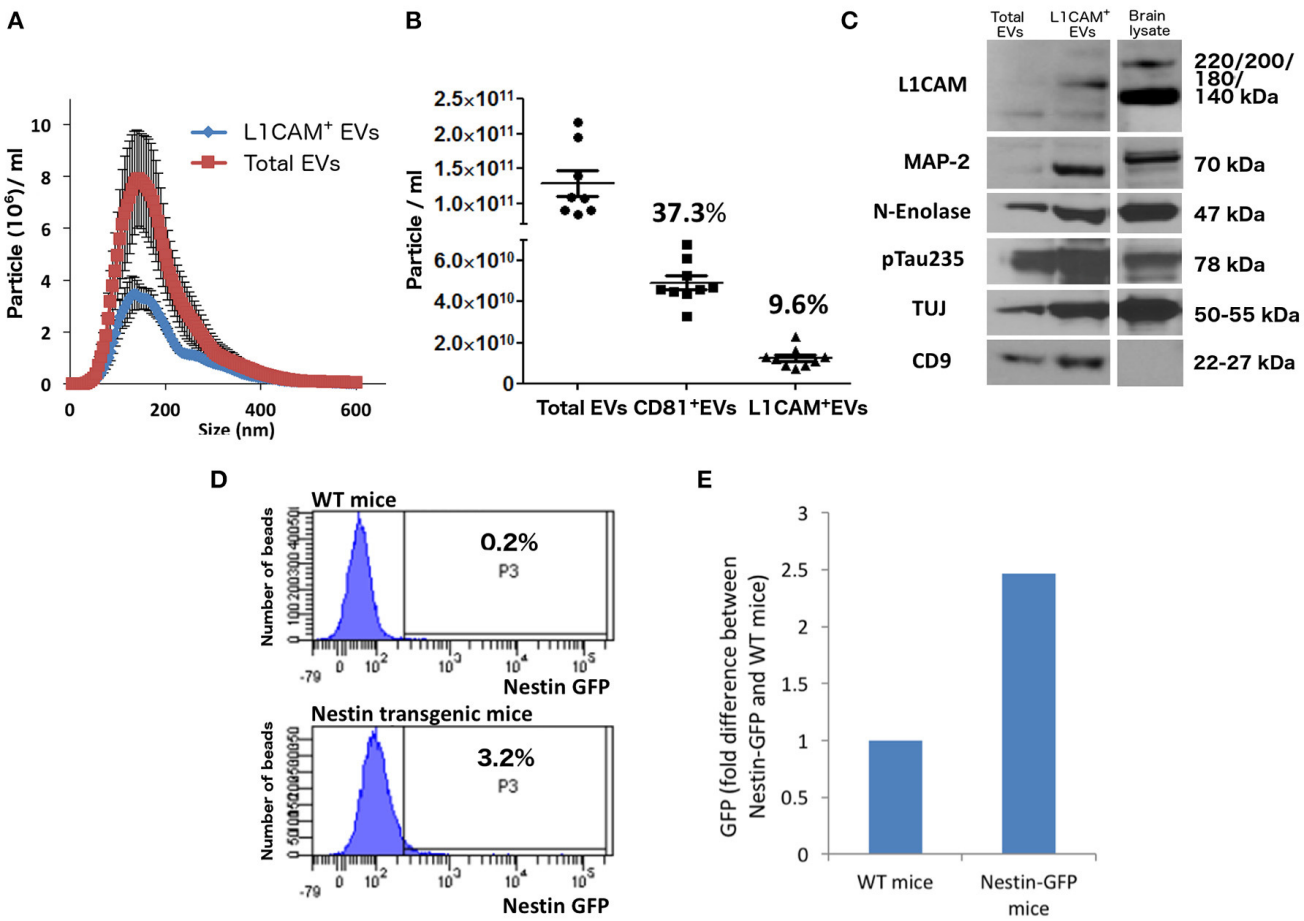
**Neuronal EVs are found in the circulation. (A)** Total EVs and L1CAM+ EVs were isolated from plasma of nine healthy volunteers and their size distribution was examined by NTA using Nanosight-NS500. Figure depicts concentration after 1:1,000 dilution for Total EVs and 1:200 dilution for L1CAM+ EVs; actual concentrations are depicted in **(B)**. **(B)** The graph shows the actual concentration of total EVs and L1CAM+ and CD81+ plasma EVs immunoprecipitated from plasma of nine healthy volunteers after adjusting for dilution. The percentages in the graph represent the ratio over total EVs. **(C)** Western blot image shows enrichment of neuronal markers (L1CAM, MAP-2, N-enolase, p-Tau235, and TUJ) in L1CAM+ EVs when compared to total EVs from a single healthy control. CD9 is a common exosomal marker present in EVs but not in the mouse brain lysate used as a positive control. An equivalent amount of EVs was loaded on the gel by adjusting the dilution of the isolates according to the EV concentration determined by NTA. **(D)** GFP levels evaluated by FACS; L1CAM+ EVs were isolated from 300 μl plasma derived from Nestin-GFP transgenic or WT mice. The EVs were conjugated with the beads and the levels of GFP were evaluated by FACS analysis. The results show the percentages of beads-antibody-EV complex that contained GFP above the detection threshold. **(E)** GFP levels evaluated by fluorescence; comparison between the levels of GFP in EVs in the samples described in **(D)** were measured by plate reader at excitation of 485 nm and emission 515 nm.

**Figure 3:**
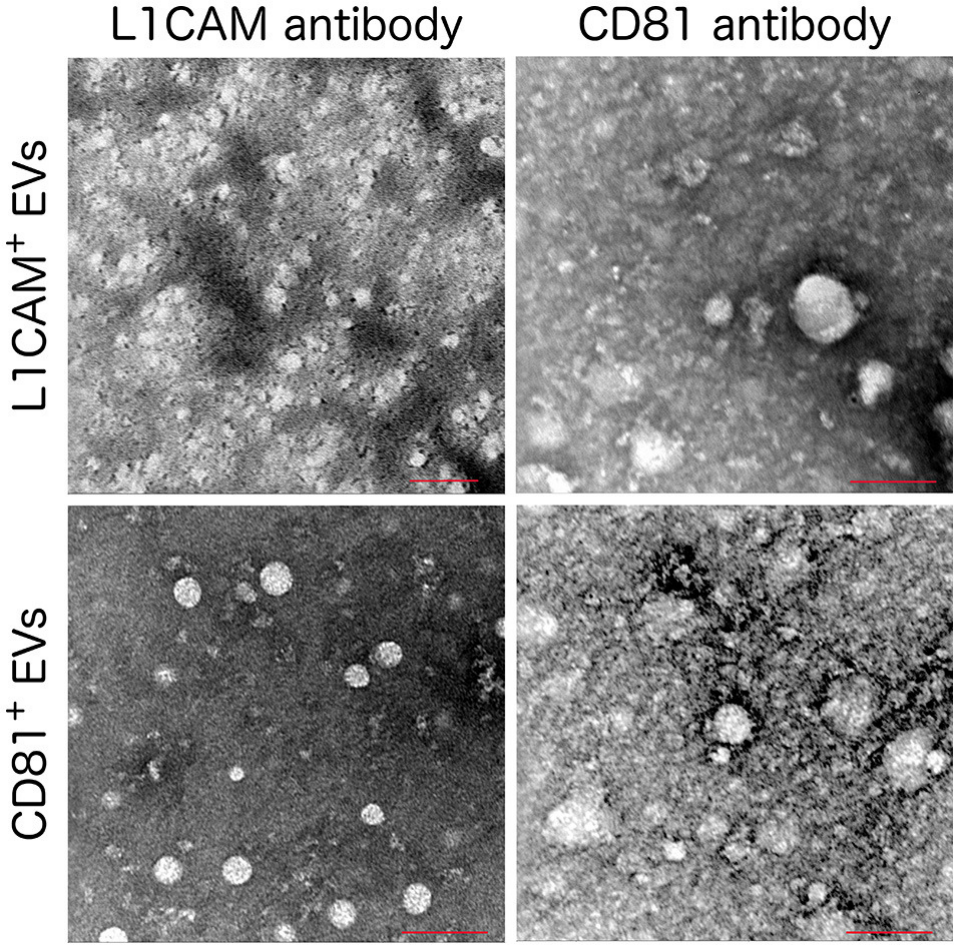
**Immuno Electron Microscopy images of L1CAM+ and CD81+ EVs**. L1CAM+ or CD81+EVs were incubated with primary human anti-L1CAM or CD81 antibody followed by a secondary antibody conjugated with 6 nm gold particle. Scale bar = 100 nm, microscope settings; 120 kV, magnification 200,000x except the L1CAM+ labeled with anti-L1CAM (top left) which used a magnification of 160000x.

The isolation of EVs enriched for neuronal origin from peripheral blood provides a new diagnostic platform that may dynamically reflect and track neuropathological changes *in vivo*. Our methodology (Figure 1) has been published repeatedly with few variations (Fiandaca et al., 2015; Goetzl et al., 2015b, 2016a,b; Kapogiannis et al., 2015). This method efficiently isolates total EVs from plasma or serum samples using a commercially available high-throughput particle precipitation method (Exoquick®; Saenz-Cuesta et al., 2015), followed by immunoprecipitation with biotinylated antibodies against neuronal surface markers to isolate sub-populations of NCAM+ or L1CAM+ EVs. Neural cell adhesion molecules NCAM and L1CAM (CD171) were selected as targets for immunoprecipitation due to their high and relatively specific expression in neural tissue and early research demonstrating high expression on exosomes derived from cultured neurons (Faure et al., 2006). Nanoparticle tracking analysis (NTA) shows that the L1CAM+ EVs have size distribution similar to total plasma EVs (Figure 2A), but their concentration is 90–95% lower (Figure 2B). To assess the success of the immunoprecipitation, we confirmed the presence of the target molecule L1CAM on EVs following immunoprecipitation by immuno-electron microscopy using antibodies against L1CAM tagged with gold particles (Figure 3). This image shows that we have isolated a range of EV sizes, the majority of which demonstrate binding of one or more L1CAM antibody-gold particle complexes on their surface in the L1CAM+ EVs, but fewer in CD81+ EVs. As expected, the presence and frequency of CD81 antibody-gold particle complexes is much stronger in CD81+ EVs.

In Figure 2C, we show that L1CAM+ EVs have higher concentration of several markers that are reasonably specific to neurons (p-tau, neuron-specific enolase, MAP2, NCAM, NFL, and L1CAM) compared to total plasma EVs. The expression of several neuronal markers in the same EV subpopulation is highly suggestive of neuronal [and, to some degree, Central Nervous System (CNS)] origin. It is important to note that CNS neurons comprise only about 0.3% of the entire population of human cells, and it is currently unknown whether their ability to secrete EVs is higher or lower than other cell types or if their contribution to peripheral blood EVs is proportional to their number.

To provide mechanistic proof for some degree of CNS neuronal origin of L1CAM+ plasma EVs, we isolated L1CAM+ EVs from pooled plasma from transgenic mice expressing plasma membrane-bound green florescent protein (GFP) on a nestin promoter that is specifically expressed in the nervous system. Our fluorescence-activated cell sorting (FACS)-type analysis showed that 3.2% of the beads incubated with pooled L1CAM+ EVs isolated from nestin promoter-GFP transgenic mice generated a suprathreshold GFP signal, as opposed to only 0.2% of the beads incubated with pooled L1CAM+ EVs isolated from WT mice (Figure 2D). To confirm this finding with a second technique, we used a plate reader at an excitation of 485 nm and an emission of 515 nm. This analysis revealed a 2.5-fold difference in the GFP fluorescent signal in pooled L1CAM+ EVs isolated from nestin-GFP transgenic mice compared to pooled L1CAM+ EVs from WT mice (Figure 2E). The current sensitivity and technical limitations of these techniques do not allow for reliable quantitative results, as a sufficient number of GFP+ L1CAM+ EVs must bind to a bead to render it detectable over the threshold for FACS-type analysis. Qualitatively, these findings do prove that a measurable portion of the L1CAM+ EVs originate from the CNS and cross or bypass the BBB to reach the systemic circulation. The mechanism by which brain EVs may cross the BBB is still unclear. There is broad evidence for EV transfer between cells, but again the specific mechanisms have not been elucidated (Chen et al., 2016).

## Assessing neuronal-origin enrichment of L1CAM+ EVs

**Figure 4:**
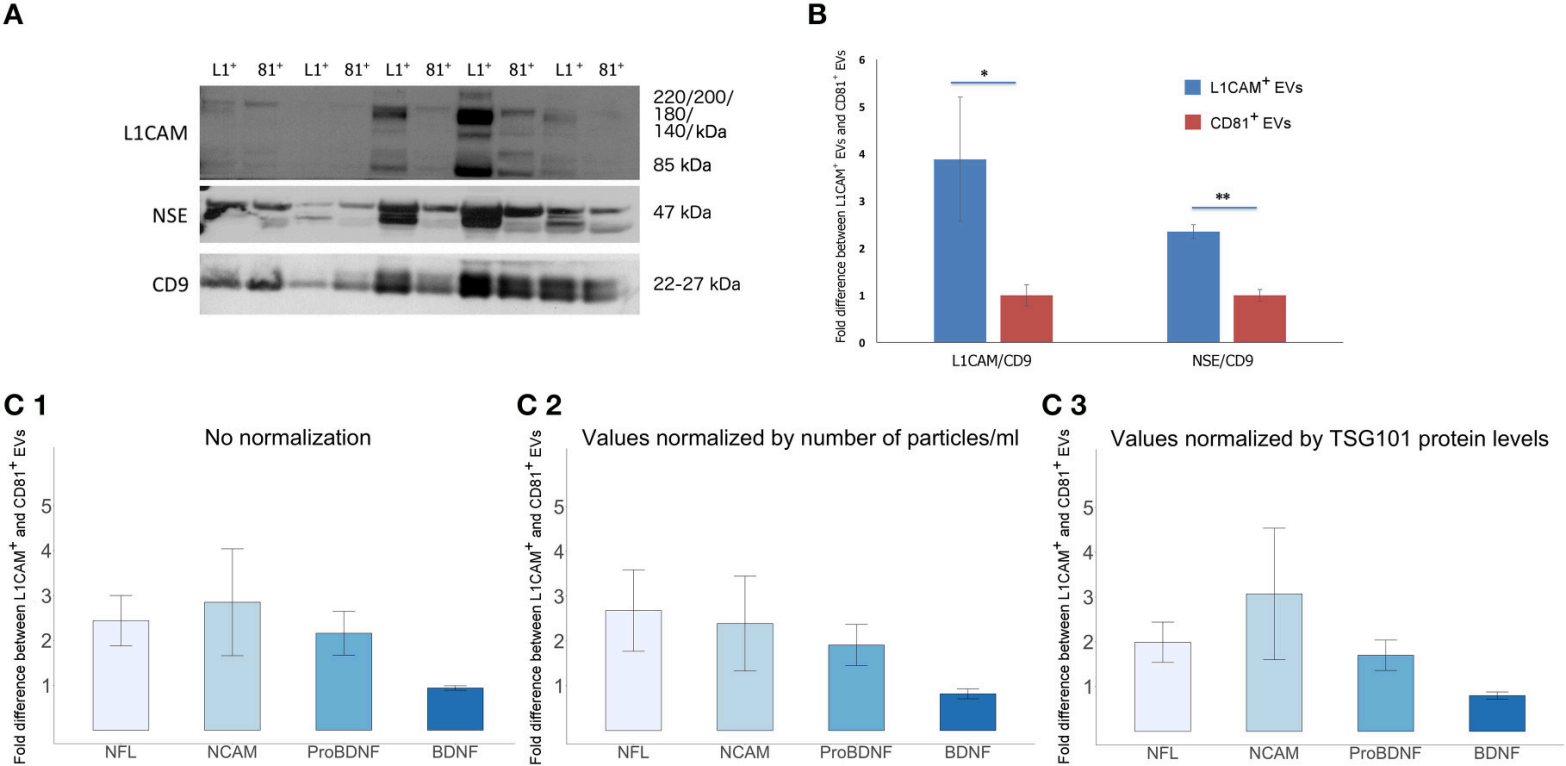
**L1CAM+ EVs are enriched for neuronal origin. (A)** L1CAM+ and CD81+ plasma-derived EVs from five healthy volunteers (out of 10 showing similar data). Western blots of L1CAM+ EVs (L1^+^) are set adjacent to corresponding CD81+ EVs (81^+^) for each individual. The membrane was stained for L1CAM (top), neuron-specific enolase (NSE; middle), and EV marker CD9 (bottom). An equivalent amount of EVs were loaded on the gel by adjusting the dilution of the isolates according to the EV concentration determined by NTA. **(B)** Enrichment of neuronal markers in L1CAM+ EVs compared to CD81+ EVs by Western blots [L1CAM+ EVs (Blue, *N* = 10); CD81+ EVs (Red, *N* = 10)]. Enrichment is expressed as a fold difference in the ratio of L1CAM or NSE over CD9 signal. ImageJ was used to determine the signal intensity of each marker. A paired *t*-test was used to determine statistical differences between L1CAM+ and CD81+ EVs, error bars represent SEM of 10 subjects. Significance ^*^p < 0.05, ^**^p < 0.0001. **(C)** Enrichment of neuronal markers in L1CAM+ EVs compared to CD81+ EVs by ELISA for neuronal markers, NFL, NCAM, BDNF, proBDNF. (1) Fold difference in protein levels in L1CAM+ EVs to CD81+ EVs: L1CAM+ EVs contain 2.44 ± 0.56 (mean ± SEM) fold more NFL, 2.85 ± 1.19-fold more NCAM, and 2.16 ± 0.49-fold more proBDNF than CD81+ EVs (*N* = 10 healthy volunteers, measured in duplicate). L1CAM+ EVs contain amounts (0.94 ± 0.05) of BDNF similar to those of CD81+ EVs. (2) Fold difference in protein levels in L1CAM+ EVs to CD81+ EVs normalized to number of EV particles/ml measured by NTA. (3) Fold difference in protein levels in L1CAM+ EVs to CD81+ EVs normalized to TSG101 protein levels measured using custom electroluminescence assay. These results show that L1CAM+ EVs contain consistently and substantially higher levels of a range of neuronal proteins compared to total and control sub-populations.

To assess the degree by which plasma derived L1CAM+ EVs are enriched for neuronal origin, we examined several neuronal markers in L1CAM+ EVs compared to EVs immunoprecipitated by an antibody to the canonical exosome surface marker CD81. For these experiments, we performed EV enrichment for L1CAM and CD81 in paired plasma samples from 10 healthy controls. The EVs isolated from plasma by CD81 immunoprecipitation represent a carefully selected control, since they have diverse cellular origins, similar to total plasma EVs, but have undergone the same experimental conditions and handling as EVs isolated by L1CAM immunoprecipitation. Levels of L1CAM, neuronal enolase (NSE), and the canonical EV marker CD9 used for normalization were measured by Western blot (WB) analysis (Figure 4A). Quantification of the WB showed that L1CAM+ EVs contain 3.88-fold higher levels of L1CAM and 2.35-fold higher levels of neuronal enolase than CD81+ EVs (Figure 4B). In addition, we used ELISA assays to measure several neuronal proteins [neurofilaments-light (NFL), NCAM, brain derived neurotrophic factor (BDNF), and pro-BDNF] in L1CAM+ and CD81+ EVs in duplicate samples. The graph depicted in Figure 4C1 shows that L1CAM+ EVs contain, on average, 2.44-fold higher levels of NFL, 2.85-fold higher levels of NCAM, and 2.16-fold higher levels of proBDNF than CD81+ EVs. There was no difference in BDNF levels between L1CAM+ EVs and CD81+ EVs (0.94-fold difference). Similar results were obtained when ELISA results were normalized either by number of EV particles/ml (Figure 4C2) or by levels of the canonical exosomal marker TSG101 (Figure 4C3). Altogether, these results consistently show that L1CAM+ EVs contain much higher levels of a range of neuronal proteins than those in total EVs and control EV sub-populations.

## EVs enriched for neuronal origin as source of biomarkers

**Figure 5:**
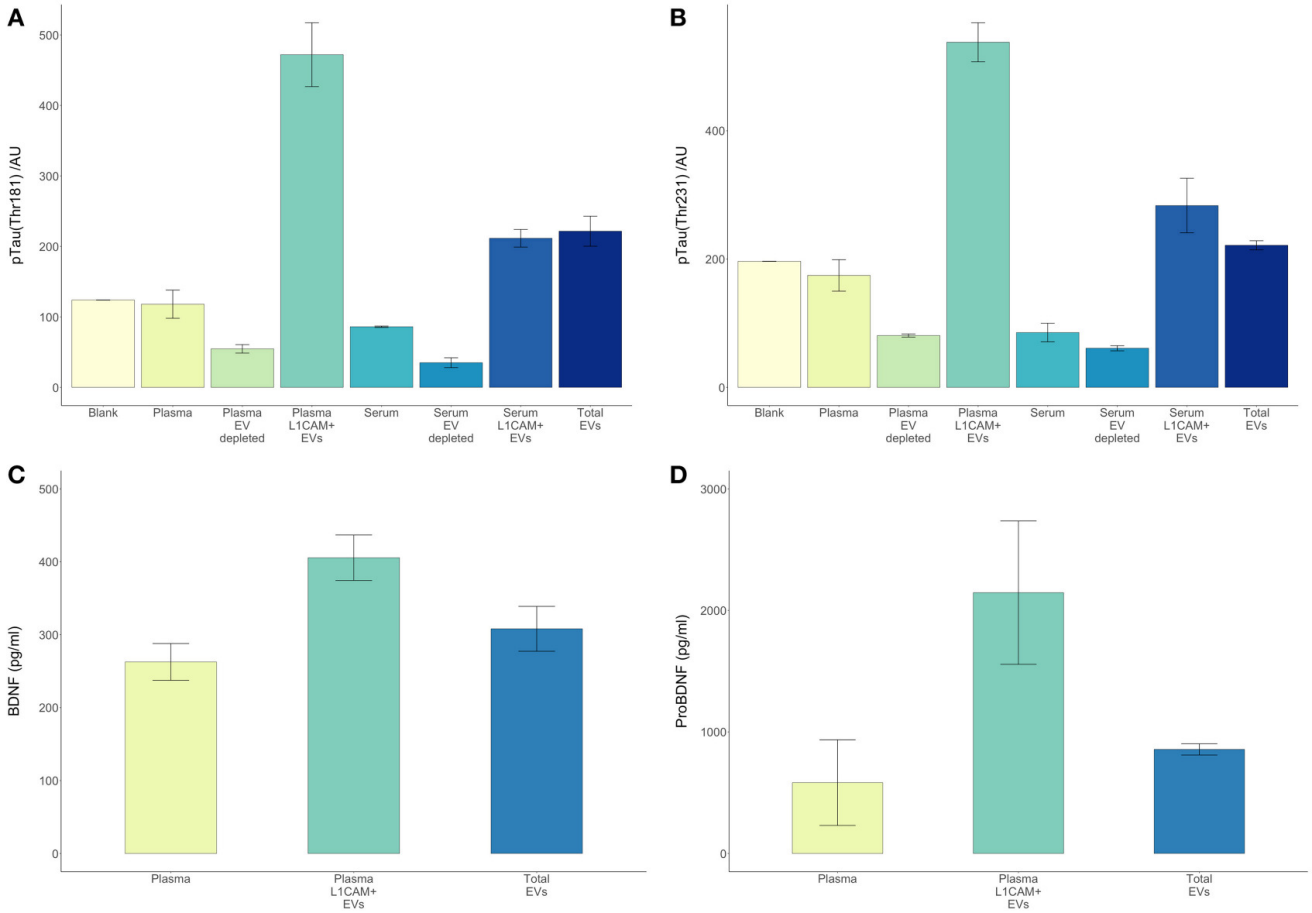
**L1CAM+ EVs offer a higher detection level for p-tau, BDNF and pro-BDNF over plasma, serum and total EVs**. For p-tau comparisons, total EVs were isolated from four plasma and serum samples from healthy volunteers followed by L1CAM immunoprecipitation. The levels of p-tau-Thr181 **(A)** and p-tau-Thr231 **(B)** are presented in the graph in L1CAM+ EVs, total EVs, plasma, serum, and in comparison, to the background signal (blank). Column bars represent the mean of four samples, error bars represent SEM. For BDNF and proBDNF comparisons, total EVs were isolated from 20 plasma samples from healthy volunteers followed by L1CAM immunoprecipitation. BDNF levels **(C)** are different depending on the type of fluid tested [*F*_(2, 57)_ = 6.868, *p* = 0.002]; its levels are higher in L1CAM + EVs compared to plasma (*p* = 0.001) and total EVs (*p* = 0.016), whereas its levels in total EVs were no different than plasma (*p* = 0.254). Similarly, proBDNF levels **(D)** are dependent on the type of fluid tested [*F*_(2, 57)_ = 4.41, *p* = 0.017]; its levels are higher in L1CAM + EVs compared to plasma (*p* = 0.007) and total EVs (*p* = 0.026), whereas its levels in total EVs were no different than plasma (*p* = 0.628).

There are several theoretical advantages to using EVs enriched for neuronal origin as a means to derive biomarkers for neurological disorders. Neuronal enrichment of EVs can improve the signal to noise ratio, increase measurement sensitivity, lower the detection threshold (by providing an extract with higher concentrations of a target molecule than plasma or total EVs), and better reflect pathophysiological processes occurring in neurons. In this setting, it is illustrative to examine the case of tau and its various phosphorylated forms, which are highly involved in the development AD pathology and very difficult to detect in plasma or serum. Here, we reproduced our previous observation that circulating levels of p-T181-tau were below detection levels in serum and plasma samples even when using a sensitive electrochemiluminescence based assay (Figure 5). However, both p-T181-tau and p-T231-tau were detected in a high concentration in both plasma and serum derived L1CAM+ EVs (Figures 5A,B). The higher levels of these tau phospho-species in plasma-derived L1CAM+ EVs than in serum-derived L1CAM+ EVs are probably due to the higher concentration of EVs in plasma compared to serum samples (Muller et al., 2014).

BDNF is a neuronal extracellular signaling protein with a prominent role in nervous system development (Murray and Holmes, 2011), learning and memory (Bekinschtein et al., 2008), and neuronal stress resistance (Marosi and Mattson, 2014). Both BDNF and its precursor protein proBDNF (Koshimizu et al., 2009) were detected in significantly and substantially higher concentrations in L1CAM+ EVs compared to plasma samples (Figures 5C,D).

Our team and others have used EVs enriched for neuronal origin in a series of case-control studies to demonstrate proof of concept for this approach in AD (summarized in Table 1). Given that intraneuronal deposits of hyper-phosphorylated tau and extracellular deposits of Aβ fibrils are neuropathological hallmarks of AD (Arnold et al., 1991; Braak and Braak, 1991; Braak and Del Tredici, 2012), we first measured total tau, p-T181-tau, p-S396-tau, and Aβ42. All marker levels other than total tau were substantially and significantly higher in AD patients compared to controls (Kapogiannis et al., 2014; Fiandaca et al., 2015). Similar differences were found for the progression from MCI to dementia (Winston et al., 2016), and in Down syndrome patients (Hamlett et al., 2016). In addition to pathogenic proteins, we turned our attention to a number of intracellular signaling molecules implicated in AD pathogenesis, such as phosphorylated insulin receptor substrate-1 (IRS-1) species, lysosomal proteins (cathepsin-D), heat shock proteins (HSP70), cellular survival factors (REST), LRP6, and others (Fiandaca et al., 2015; Goetzl et al., 2015b; Kapogiannis et al., 2015). These studies demonstrated that certain EV proteins (p-T181-tau, p-S396-tau, Aβ42, and pTyr- and pSer-IRS-1) achieved impressive classification accuracy between patients and controls (Fiandaca et al., 2015; Kapogiannis et al., 2015). Changes in synaptic proteins were also reported in a small cohort of AD and Frontotemporal Dementia patients, and there was some evidence that synaptopodin, synaptotagmin, and synaptophysin may track disease severity due to their association with cognitive performance (Goetzl et al., 2016a). A study using a similar immunoprecipitation technique that also targeted L1CAM showed a highly significant increase in alpha-synuclein in L1CAM+ EVs in Parkinson's disease (PD) patients compared to controls, which also correlated with disease severity (Shi et al., 2014). These findings highlight the value of enrichment for L1CAM for neurodegenerative diseases, but also provide an example of the potential for plasma-derived EV subpopulations to serve as a biomarker discovery platform for other conditions. The accumulated results of these studies raise hope that a EV-based blood test may allow the diagnosis of AD and other neurodegenerative diseases at the crucial preclinical stage. Ongoing studies conducted in larger datasets, particularly in a large cohort from the Baltimore Longitudinal Study of Aging, aim to replicate and validate these initial successes and expand the diagnostic potential of neural origin EVs.

**Table 1:** **Published studies using L1CAM+ EVs as a source of biomarkers for various diseases**.

**Disease**	**Participants**	**Metabolites**	**References**
Down syndrome (DS)	47 DS; 37 controls	Aβ42	Hamlett et al., 2016
		P-T181 TAU	
		P-S396 TAU	
Fetal alcohol syndrome (FAS)	20 pregnant women (10 EtOH users; 10 non-users)	HSF1	Goetzl L. et al., 2016
		BCL-XL	
		REST	
Preclinical prediction of AD	20 AD; 18 preAD (Cognition normal)	Aβ42	Abner et al., 2016
		P-T181 TAU	
		Cathepsin D	
		REST	
		Neurogranin	
AD	24 AD; 24 control	LRRP	
		HSP1	Goetzl et al., 2015a
Preclinical prediction of AD	16 AD; 16 preAD	REST	
AD	26 AD; 26 control	P-S312-IRS-1	Kapogiannis et al., 2015
Preclinical prediction of AD	22 AD; 22 preAD	P-panY- IRS-1	
AD	26 AD; 26 control	Cathepsin D	
		LAMP1	Goetzl et al., 2015b
		Ubiquitin	
Preclinical prediction of AD	20 AD; 20 preAD	HSP70	
AD	57 AD; 57 control	Aβ42 (0.001)	
		P-T181 TAU	Fiandaca et al., 2015
Preclinical prediction of AD	24 AD; 24 preAD	P-S396 TAU	
AD	24 AD; 28 control	Synaptotagmin	Goetzl et al., 2016a
		Synaptopodin	
		Synaptophysin	
		Neurogranin	
		GAP43	
AD	30 AD; 20 controls	REST	Winston et al., 2016
		Neurogranin	
		Aβ42	
		P-T181 TAU	
		P-S396 TAU	
PD	267 PD; 215 controls	α-Synuclein	Shi et al., 2014

## Unique protein signatures of plasma EV subpopulations

**Figure 6:**
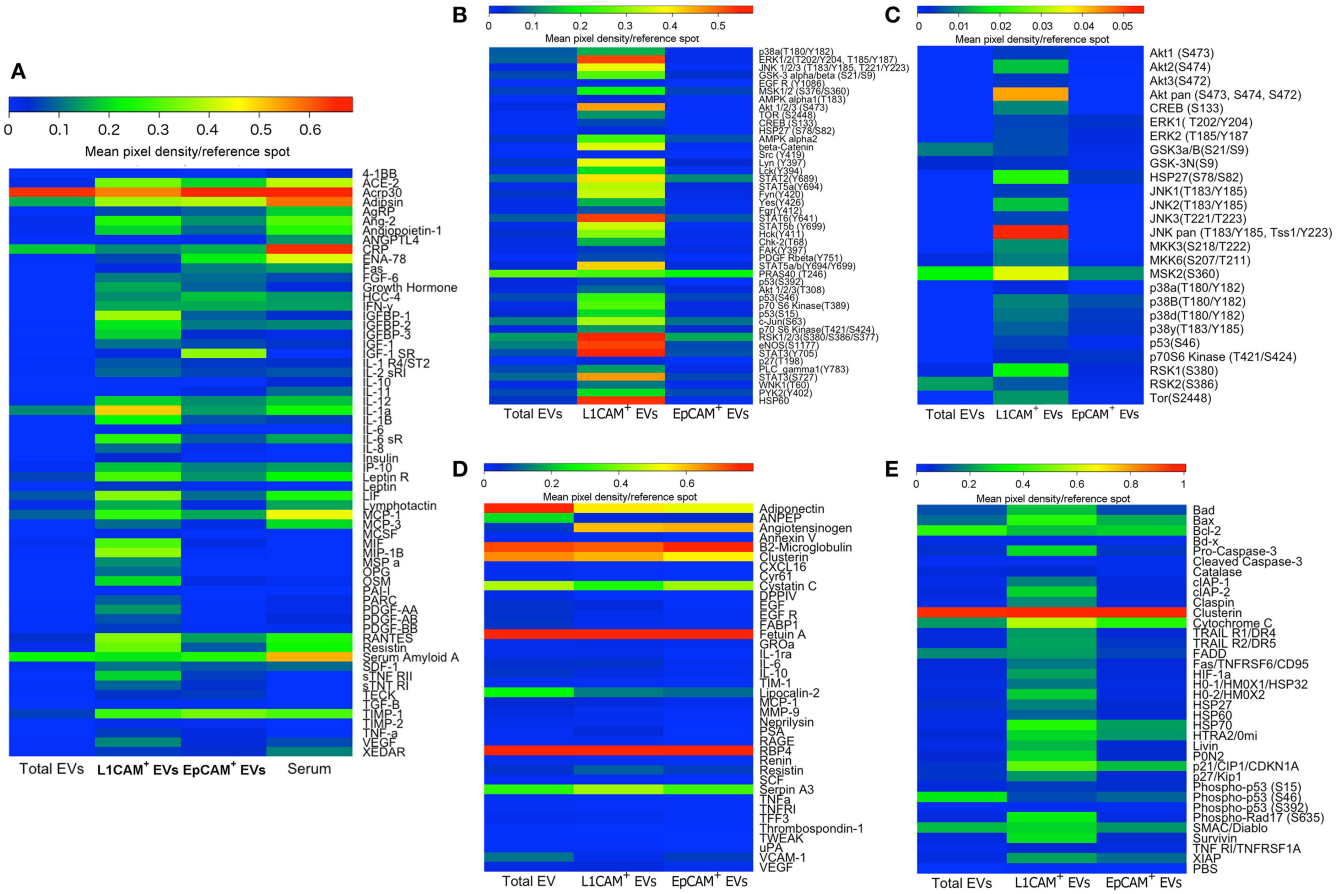
**L1CAM+ EVs contain a distinct and interesting protein signature**. Total EVs were isolated from plasma from four healthy volunteers and subsequently immunoprecipitated with L1CAM or EpCAM antibodies. The samples from the four subjects were then pooled to generate a sufficient amount of protein for five membrane antibody arrays [Human Obesity Antibody Array **(A)**, Human Phospho-Kinase Antibody Array **(B)**, MAPK Antibody Array **(C)**, Human Kidney Biomarker Antibody Array **(D)**, and Human Apoptosis Antibody Array **(E)**]. An equivalent amount of protein per sample type was loaded to each spot. The levels of different proteins were calculated by densitometric analysis and normalized to the reference proteins present on each membrane. Results are depicted as heatmaps ranging from zero to maximum value and scaled according to the color bars.

To further characterize distinct subpopulations of immunoprecipitated EVs, we isolated L1CAM+ EVs and EVs enriched for epithelial origin using epithelial cell adhesion molecule EpCAM (EpCAM+ EVs; Taylor and Gercel-Taylor, 2008; Tauro et al., 2013). Taking care to load an equivalent concentration of EVs as determined by NTA, we then compared their protein profiles directly and with total EVs and serum using several commercially available membrane arrays (Figure 6). These arrays included extracellular and intracellular proteins involved in metabolic processes and obesity (Figure 6A), phospho-kinases (Figure 6B), mediators of kidney function (Figure 6C), the MAPK pathway (Figure 6D), and apoptosis pathways (Figure 6E). Collectively, these arrays include multiple key intracellular and extracellular mediators, hormone receptors, kinases, trophic, anti- and pro-apoptotic factors, and intracellular energetic sensors, which may be suitable biomarkers for various disease states. The original membrane array blots and a table summarizing all 207 proteins measured are presented in Supplemental Figures 1–5 and Supplemental Table 1, and are also presented in heat map format for ease of comparison between EV types. It is evident from these results that L1CAM+ and EPCAM+ EVs have distinct protein profiles. For example, while HIF1, HSP27, and leptinR are higher in L1CAM+ EVs, phospho-p53, IGF1R, and ENA-78 are higher in EPCAM+ EVs. Interestingly, the L1CAM+ EVs contain much higher levels of many proteins that reflect cellular energetic status (e.g., mTOR), are downstream effectors of insulin signaling (e.g., Akt), and regulate metabolism (e.g., the Leptin receptor). This shows that even though the selection of L1CAM+ EVs was made based on their presumed neural origin, the resulting EV population reflects the highly active metabolism of their cells of origin. This suggests that L1CAM+ EVs may be a reliable source for a “liquid biopsy” to study metabolic processes. Dietary and other metabolism-based intervention studies currently include outcome measures such as biometric measures of obesity or circulating levels of metabolites or hormones, which typically reflect whole-body metabolic status. Unfortunately, the broadness of these measurements limits their potential to assess organ and system-specific responses such as changes in brain metabolism. Biomarkers found in L1CAM+ EVs that more closely reflect brain metabolism may enable us to study metabolic abnormalities in disease states such as AD, and can be used as potential outcomes in therapeutic trials.

## Current challenges and future perspectives

All cell types secrete EVs into the circulation, and our recent results as well as those of other labs (Table 1) demonstrate that the idea of utilizing cell-specific EVs as a platform for a liquid biopsy holds great promise. The EV isolation method currently employed in our laboratory was developed by Edward J. Goetzl, and generates a distinct subpopulation of plasma EVs containing a much higher concentration of neuronal proteins than total EVs and control EV sub-populations. This enrichment for neuronal origin opens a unique “window” into the brain, but a critical limitation for all antibody-based methods is that they are dependent on the quality of the antibody as well as on the cell-type specificity of the surface marker being used for isolation. For example, L1CAM is highly expressed in neurons, but is also expressed at low levels in many other cell types (e.g., lymphocytes). Many of the currently used cellular markers have been developed for histopathological studies, in which they are used to compare neurons to other cells in the same tissue. Further, research and discovery of cellular surface markers that are cell-specific even when compared to the entire organism is needed for the development of a true “liquid biopsy.”

## Conclusions

To spearhead therapeutic discovery, there is a pressing need to establish biomarkers for AD and other neurodegenerative diseases that are non-invasive, widely available, and inexpensive. Blood-based biomarkers meet these standards, but measures in the liquid phase of plasma have failed to show adequate sensitivity and specificity for clinical applications. We have shown the potential of peripheral blood EVs enriched for neuronal origin to generate such biomarkers in several clinical studies, and further work is under way to improve the balance between specificity and yield. We are hopeful that this line of research will provide biomarkers with diagnostic performance akin to a true brain “liquid biopsy” to advance the field of neurodegenerative diseases. Moreover, we are hopeful that EV-based biomarkers will show dynamic and mechanism-specific response to experimental treatments, allowing their use as therapeutic-response biomarkers in clinical trials for AD and other neurodegenerative diseases.

## Materials and methods

### Two step isolation of EV subtypes

EVs were isolated from 0.5 ml of frozen human plasma containing EDTA or serum collected in serum separator tubes (SST). Samples were defrosted and each sample received 15 μl of Thrombin (System Biosciences, Inc., Mountainview, CA) followed by a 30 min incubation at room temperature (RT, ~23°C). After the addition of 485 μl of Dulbecco's calcium- and magnesium-free salt solution containing three times more than the suggested concentrations of protease inhibitor cocktail (Complete Tablets Easy pack, Roche Applied Sciences, Inc., Indianapolis, IN) and phosphatase inhibitor cocktail (Pierce Halt, Thermo-Fisher Scientific, Inc., Rockford, IL), samples were mixed, left for 5 min at RT and then centrifuged at 4,000 × g for 20 min at 4°C. Supernatants were transferred to fresh tubes and gently mixed by inversion after addition of 252 μl of Exoquick® exosome solution (System Biosciences, Inc., Mountainview, CA). Suspensions with Exoquick® were incubated for 60 min at 4°C to precipitate total EVs (Peterson et al., 2015) and then centrifuged at 1,500 × g for 20 min at 4°C. Supernatants were discarded after centrifugation and the pellet containing EVs was re-suspended in 0.5 ml of Ultra-pure distilled water (Invitrogen-Thermo-Fisher Scientific, Inc., Rockford, IL, USA) containing three times the suggested concentrations of protease and phosphatase inhibitors. To enrich for EVs containing L1 Cell Adhesion Molecule (L1CAM), suspensions were incubated for 1 h at 4°C with 4 μg of mouse anti-human CD171 (L1 cell adhesion molecule [L1CAM]) biotinylated antibody (CD171, clone 5G3, eBioscience, San Diego, CA) in total volume of 50 μl of 3% BSA (1:3.33 dilution of Blocker BSA 10% solution in PBS [Thermo Scientific, Rockford, IL, USA]) per tube with mixing, followed by addition of 15 μl of streptavidin-agarose Ultralink resin (Thermo Scientific, Rockford, IL, USA) in total volume of 40 μl of 3% BSA and incubation for 30 min at 4°C with continuous mixing. In this step for isolating different EV subpopulations, L1CAM antibody was replaced with biotinylated antibody against either CD81 (CD81-biotin, Ancell, Bayport, MN), EpCAM (MA5-12150, Thermo Scientific, Rockford, IL, USA), or no antibody was added. After centrifugation at 200 × g for 10 min at 4°C and removal of the supernatant, each pellet was re-suspended in 200 μl of 0.1 M glycine-HCl by mixing for 10 s and centrifuged at 4,500 × g for 10 min at 4°C to detach L1CAM+EVs from the bead-antibody complex. Supernatants were then transferred to clean tubes containing 25 μl of 10% BSA and 15 μl of 1 M TRIS-HCl and mixed. To lyse EVs, each tube received 260 μl of mammalian protein extraction reagent (M-PER; Thermo Scientific, Rockford, IL, USA), containing three times the suggested concentrations of protease and phosphatase inhibitors and went through 2 freeze thaw cycles. Final suspensions containing EV proteins were stored at −80°C.

### Nanoparticle tracking analysis (NTA)

An EV suspension aliquot (10 μl of vesicles in 90 μl PBS) collected after addition of glycine and before lysis with M-PER was diluted to final dilution of 1:200 in PBS. The mean diameter (nm) and concentration (particles/ml) of EVs was determined using the Nanosight NS500 system with a G532 nm laser module and NTA 3.1 nanoparticle tracking software (Malvern Instruments, Malvern, UK).

### Electron microscopy

Following immunoprecipitation, L1CAM+ and CD81+ EVs were neutralized and fixed in 4% paraformaldehyde for 10 min and subsequently quenched with 50 mM glycine for 3 min. After washing with phosphate buffered saline, vesicles were adsorbed to carbon coated transfer grids (Electron Microscopy Sciences, Hatfield, PA) and incubated for 30 min with 3% bovine serum albumin. Grids and vesicles were incubated with mouse anti-human CD171 (L1CAM; clone 5G3, eBioscience, San Diego, CA) or mouse anti-human CD81 (clone 5A6, Santa Cruz Biotechnology, sc-23962) antibody for 1 h, washed in blocking buffer and further incubated with IgG-gold (6 nm) conjugates (6 nm Colloidal Gold-AffiniPure Goat Anti-Rabbit IgG, Jackson Laboratory, West Grove, PA). After thorough washing, grids were finally incubated for 30 s in uranyl acetate 4% for negative staining. They were subsequently visualized at 120 kV in the Zeiss LSM 200 Transmission Electron Microscope (Zeiss, Jena, Germany).

### Dual fluorescence cre-lox mouse model (Nestin-GFP)

Mouse lines were purchased from Jackson Laboratories (ROSA mT/mG, stock number 007676; Nestin-Cre, stock number: 003771). Cre-positive male mice were bred to homozygous ROSA mT/mG females to generate mice with tissue-specific expression of GFP. ROSA mT/mG transgenic mice are a model of a cell membrane-targeted, two-color fluorescent Cre system; expressing red fluorescence localized to cell membrane in widespread cells/tissues prior to Cre recombinase exposure, and green fluorescence localized to cell membrane in Cre recombinase-expressing cells. Engineered Nestin-GFP mice express Cre recombinase in central and peripheral nervous system, including neuronal and glial cell precursors. Following exposure to Cre recombinase and excision of the tdTomato expression cassette, the rearranged mT/mG transgene converts to the expression of GFP (enhanced green fluorescent protein) under control of the nestin promoter thus exhibiting membrane-localized green fluorescence (including EV membranes). All mice were on a C57BL/6 background. Mice were given *ad libitum* access to food and water. All animal experiments were approved by the Johns Hopkins Animal Care and Use Committee and performed with strict adherence to their guidelines.

Blood was obtained via non-terminal cheek bleed. Total volume of plasma (300 μl) was collected in 21 blood draws over a nine-month period from seven Nestin-GFP and two WT (C57BL/6) mice and was used (after pooling to generate sufficient amounts) for L1CAM+ EV isolation as described. Twenty microliters of 4.5 μm Dynabeads were incubated with 2 μg L1CAM antibody for 2 h at 4°C and afterwards washed twice with PBS. The L1CAM antibody coated beads were added to the isolated EVs and the samples were incubated overnight at 4°C on rotating mixer. The next day EVs-beads complex was washed once with PBS and then incubated with CD63-PE (PE anti-mouse CD63, #143903, BioLegend, San Diego, CA, USA) antibodies for 1 h at room temperature. After a subsequent wash the sample was analyzed by flow cytometry (FACS Canto II BD, Franklin Lakes, NJ). The data and figures were generated with FlowJo software (Tree Star, Inc.); a complete analysis is provided in Supplemental Figure 6.

### Membrane antibody arrays

To generate the data depicted in Figure 6, we isolated total EVs from plasma samples of four healthy subjects and proceeded with alternative immunoprecipitation for L1CAM and EpCAM. To generate a sufficient amount of protein for the arrays (~300 μg), we pooled the EV preparations from the four subjects. We used the Proteome Profiler Human Phospho-Mitogen-activated Protein Kinase (MAPK) Antibody Array (Catalog # ARY002B), Human Kidney Biomarker Antibody Array (Catalog # ARY019), Human Phospho-Kinase Antibody Array (Catalog # ARY003B), Human Apoptosis Antibody Array (Catalog # ARY009) by R&D Systems Inc. (Minneapolis, MN), and the Human Obesity Antibody Array (Catalog # ab169819) by Abcam (Cambridge, MA). These antibody-pair-based assays are analogous to ELISA, but use a membrane as a substrate rather than a plate. Capture antibodies are spotted on a membrane with each pair of spots representing a different analyte. After samples, paired biotinylated detector antibodies and Streptavidin-Horseradish Peroxidase and chemiluminescent detection reagents were added. The arrays were analyzed semi-quantitatively using ImageJ and each metabolite was normalized to the levels of positive control references on each membrane. For the depicted phospho-molecules, these arrays assess the levels of site-specific phosphorylation using phosphorylation site-specific biotinylated detector antibodies. To allow direct comparison between pooled preparations of total EVs, L1CAM+ EVs, and EpCAM+ EVs, we loaded an equivalent amount of vesicles for each EV type after measuring their concentration by NTA and diluting appropriately. To visualize results and facilitate comparisons, we generated color-coded heat maps of the density, scaled differently for each array according to the corresponding color bar, and depicting the different analytes in rows and the preparations in columns.

### ELISA and western blot quantification of the EV proteins

To generate the data depicted in Figure 4C, we isolated total EVs from plasma samples of 10 healthy subjects and proceeded with alternative immunoprecipitation for L1CAM and CD81. Phosphorylated tau residues (Thr181 and Thr231) were quantified using MSD electroluminescence assays (Meso Scale Diagnostics, Rockville, MD). The general marker for EVs TSG101 was quantified using custom electroluminescence assays developed in our laboratory using MSD GOLD Streptavidin (Meso Scale Diagnostics, Rockville, MD) coated plates and the following reagents: capture antibody was Anti-TSG101 (ab133586, Abcam, Cambridge, MA) detection was anti-TSG101 (H00007251-B01P, Novus Biologicals LLC, Littleton, CO) and recombinant protein was human TSG101 (H00007251-P01, Novus Biologicals LLC, Littleton, CO). The capture antibody was biotinylated prior to plate coating using EZ-Link™ Sulfo-NHS-LC-Biotin, No-Weigh™ Format (Thermo Fischer Scientific, Waltham, MA). The plates were read with the MESO QuickPlex SQ 120 imager (Meso Scale Discovery) using the MSD discovery workbench Software 4.0 (Meso Scale Discovery). EV suspension samples for measurement of phosphorylated tau species were undiluted (25 μl), and for TSG101 10 μl of sample was diluted with sample buffer to a final volume of 25 μl.

ELISAs for Neurofilament light (NFL) [Uman Diagnostics AB, Umea, Sweden (distributed by IBL International)] NCAM (ELH-NCAM1, RayBiotech, Inc., Norcross, GA), BDNF (BDNF Emax® ImmunoAssay System, Promega, cat. #G7611) and proBDNF [Pro BDNF (Human, Mouse, Rat) ELISA Kit, Aviscera Bioscience, cat#s SK00752-08 and SK00752-09] and Human Enolase 2/Neuron specific enolase (R&D Systems Inc., Minneapolis, MN) were run following the manufacturers' instructions. For BDNF and pro-BDNF measurements, samples were diluted by 1:2. All kit components and samples were brought to room temperature prior to use. EV protein suspensions were spun at 3,000 × g for 5 min before adding them to the wells containing capture antibodies. Samples were undiluted and the recommended volume was added to each ELISA assay. After incubation and washing, the detection antibody was added. The plate was then incubated for the recommended time and after repeated washing Avidin-HRP solution was added to the wells. After incubation and a final wash, the substrate solution was added to the wells. Following a final incubation step, stop solution was added to the plate and the absorbance was read at 450 nm with the Synergy H1 microplate reader (BioTek, Winooski, VT, USA). Protein concentrations were calculated from the standard curves with GEN5 2.04 software using a 4PL fit.

The maximum volume of EV samples per well or volume, adjusted to load an equivalent number of particles per sample, were loaded onto 4–12% NuPAGE Bis-Tris Mini gels gel (Invitrogen, Life Technologies Corporation, Grand Island, NY, USA). These were separated at 150 V for 1 min, and detected by western blot using monoclonal antibodies against L1 cell adhesion molecule [L1CAM (C-2), sc-514360], Neural cell adhesion molecule [NCAM (ERIC-1), sc-106], MAP-2(C-2, sc-390543), pTau-Ser235(sc-101812) and CD9 (H-110, sc-9148), CD81(5A6, sc-23962) [Santa Cruz Biotechnology, Inc., Dallas, TX], Neuronal enolase (NSE, MAB324, EMD Millipore, Billerica, MA), Anti-beta III Tubulin antibody (TUJ-1, ab18207), Flotilin-1(Flot-1, ab133497) [Abcam, Cambridge, MA].

## Author contributions

MM designed and performed some experiments, ELISA assays, western blots, and exosome isolations. She wrote parts of the manuscript and generated some of the figures. EE designed and performed some experiments, isolated EVs from transgenic mice expressing GFP on a Nestin promotor, measured number and size of EVs on Nanosight, and ran ELISA assays. He wrote parts of the manuscript and generated some of the figures. JW, generated electron microscopy images, ran ELISA assays, participated in the experiment that included transgenic mice expressing GFP on a Nestin promotor. He wrote parts of the manuscript and generated some of the figures. SB conducted experiments involving protein arrays and wrote parts of the manuscript. ML performed western blots and exosome isolations and wrote parts of the manuscript. JT performed western blots, exosome isolations and ELISA assays, and wrote parts of the manuscript. EG helped design the experiments, analyzed the data, and edited the manuscript. DK oversaw the design and conduct of all experiments and data analysis and wrote extensive parts of the manuscript.

### Conflict of interest statement

The authors declare that the research was conducted in the absence of any commercial or financial relationships that could be construed as a potential conflict of interest.

## Supplementary material

Supplemental Figure 1 **Original enhanced chemiluminescence (ECL) signal on film for Human Obesity Antibody array (Figure 6A)**.

Supplemental Figure 2 **Original enhanced chemiluminescence (ECL) signal on film for Human Phospho-Kinase Antibody Array (Figure 6B)**.

Supplemental Figure 3 **Original enhanced chemiluminescence (ECL) signal on film for Human MAPK Array (Figure 6C)**.

Supplemental Figure 4 **Original enhanced chemiluminescence (ECL) signal on film for Human Kidney Biomarker Antibody Array (Figure 6D)**.

Supplemental Figure 5 **Original enhanced chemiluminescence (ECL) signal on film for Human Apoptosis Antibody Array (Figure 6E)**.

Supplemental Figure 6 **Flow cytometry analysis of L1CAM+ EVs isolated from Nestin-GFP mice and WT mice**.

Supplemental Table 1 **Raw signal (pixel density) readings from densitometric analysis performed by ImageJ software and used to create heatmaps in Figures 6A–E**.
