# Supplementary figures and images for: Plasma Extracellular Vesicles Enriched for Neuronal Origin: A Potential Window into Brain Pathologic Processes
Source: Front Neurosci. 2017 May 22;11:278. doi: 10.3389/fnins.2017.00278 (PMC5439289; doi:10.3389/fnins.2017.00278)

2 min

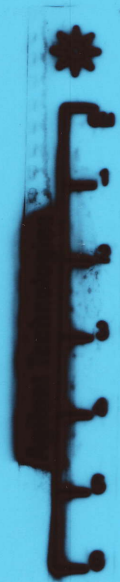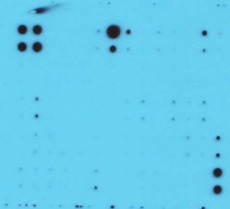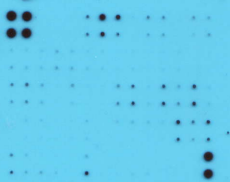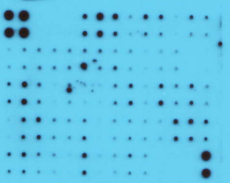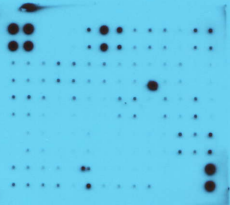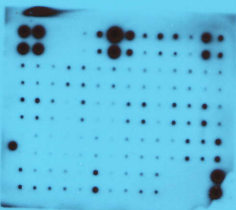

5sec

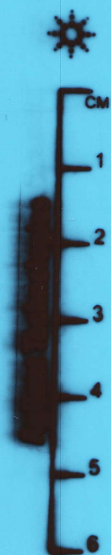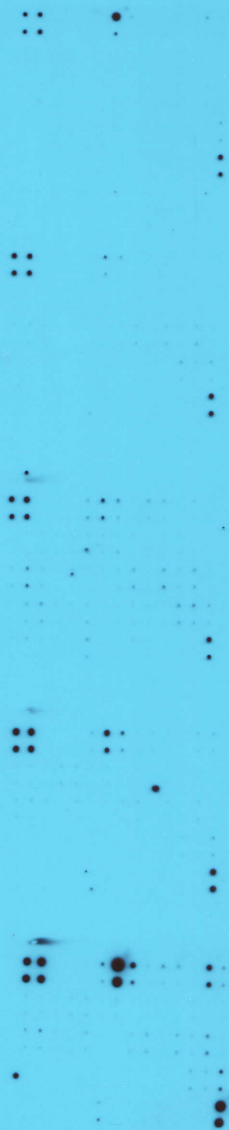

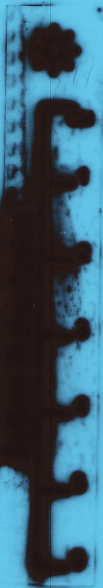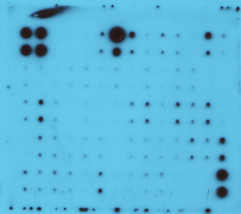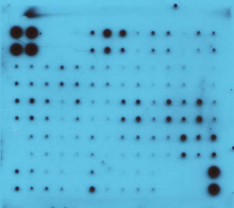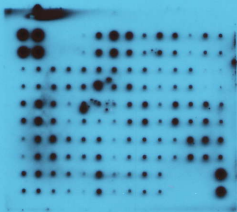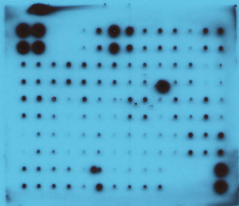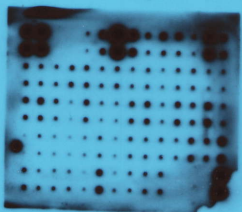

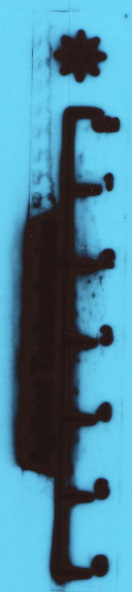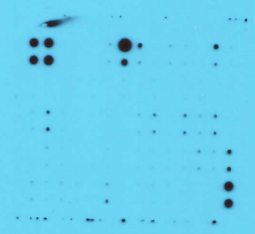

T

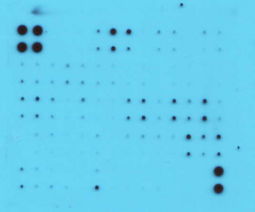

S<sub>2</sub>

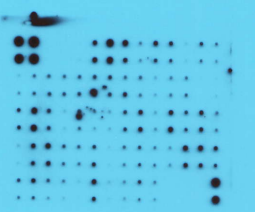

L<sub>1</sub>

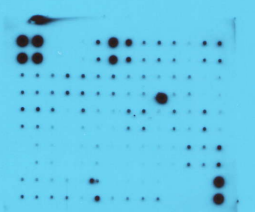

E<sub>0</sub>

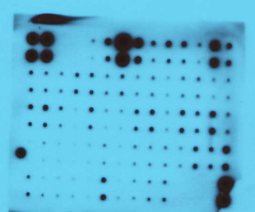

S<sub>em</sub>

Supplement: Supplemental Figure 1 — Original enhanced chemiluminescence (ECL) signal on film for Human Obesity Antibody array (Figure 6A). [file Image1.PDF]

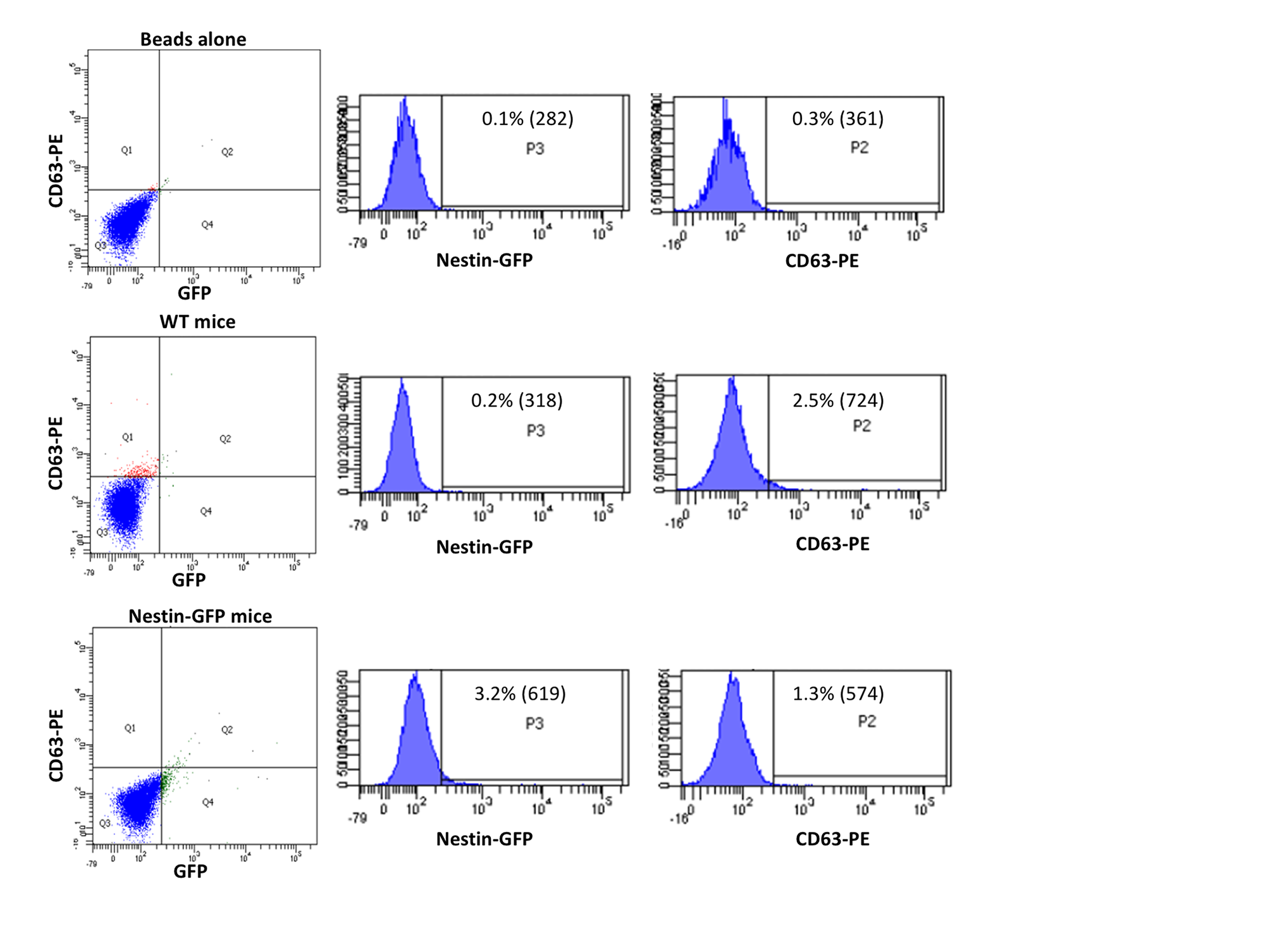

Supplement: Supplemental Figure 6 — Flow cytometry analysis of L1CAM+ EVs isolated from Nestin-GFP mice and WT mice. [file Image6.TIFF]
