# Supplementary material for: Plasma Extracellular Vesicles Enriched for Neuronal Origin: A Potential Window into Brain Pathologic Processes
Source: Front Neurosci. 2017 May 22;11:278. doi: 10.3389/fnins.2017.00278 (PMC5439289; doi:10.3389/fnins.2017.00278)
Supplement: Supplemental Figure 2 — Original enhanced chemiluminescence (ECL) signal on film for Human Phospho-Kinase Antibody Array (Figure 6B). [file Image2.PDF]

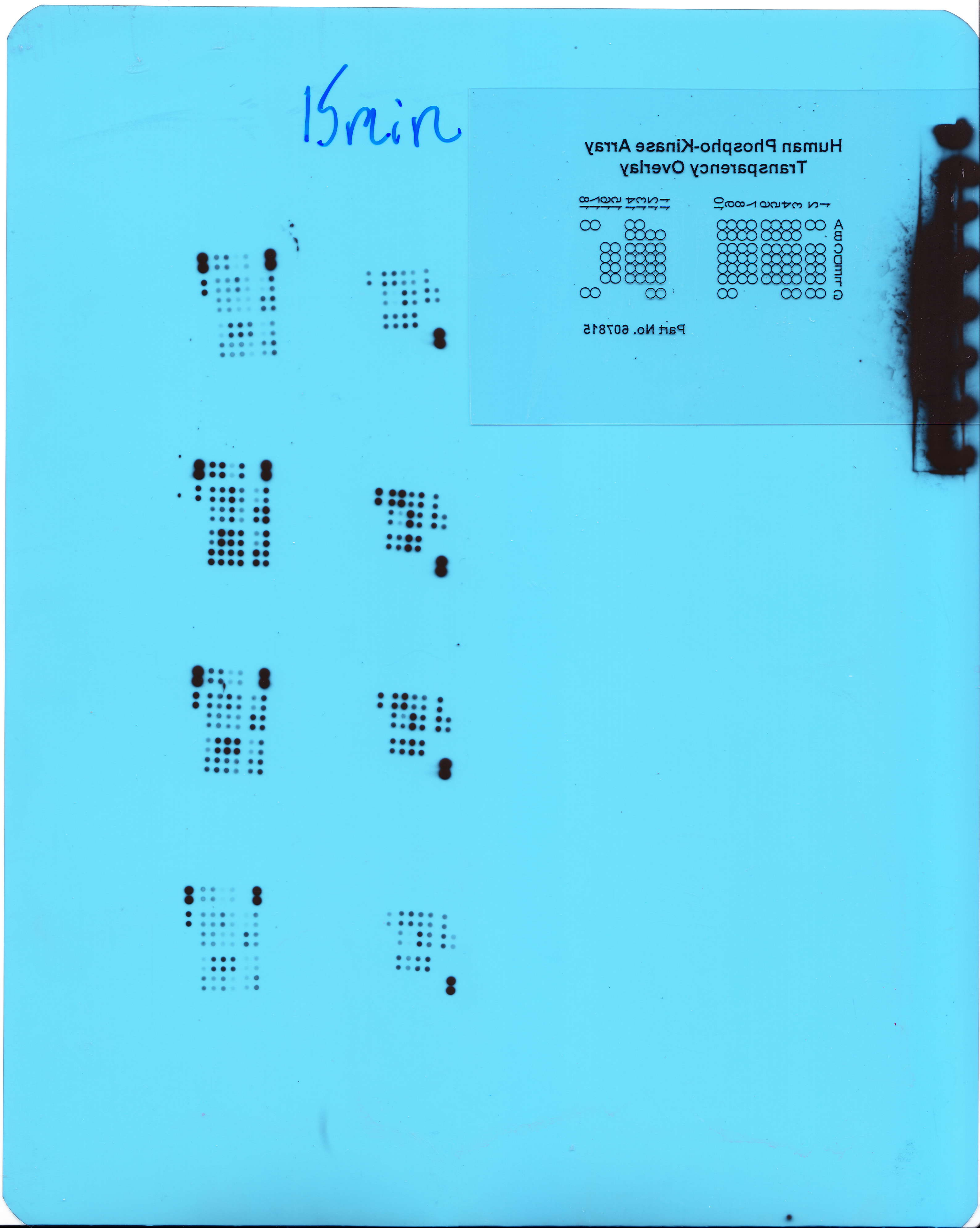
15min
Human Phospho-Kinase Array
Transparency Overlay
Part No. 607815

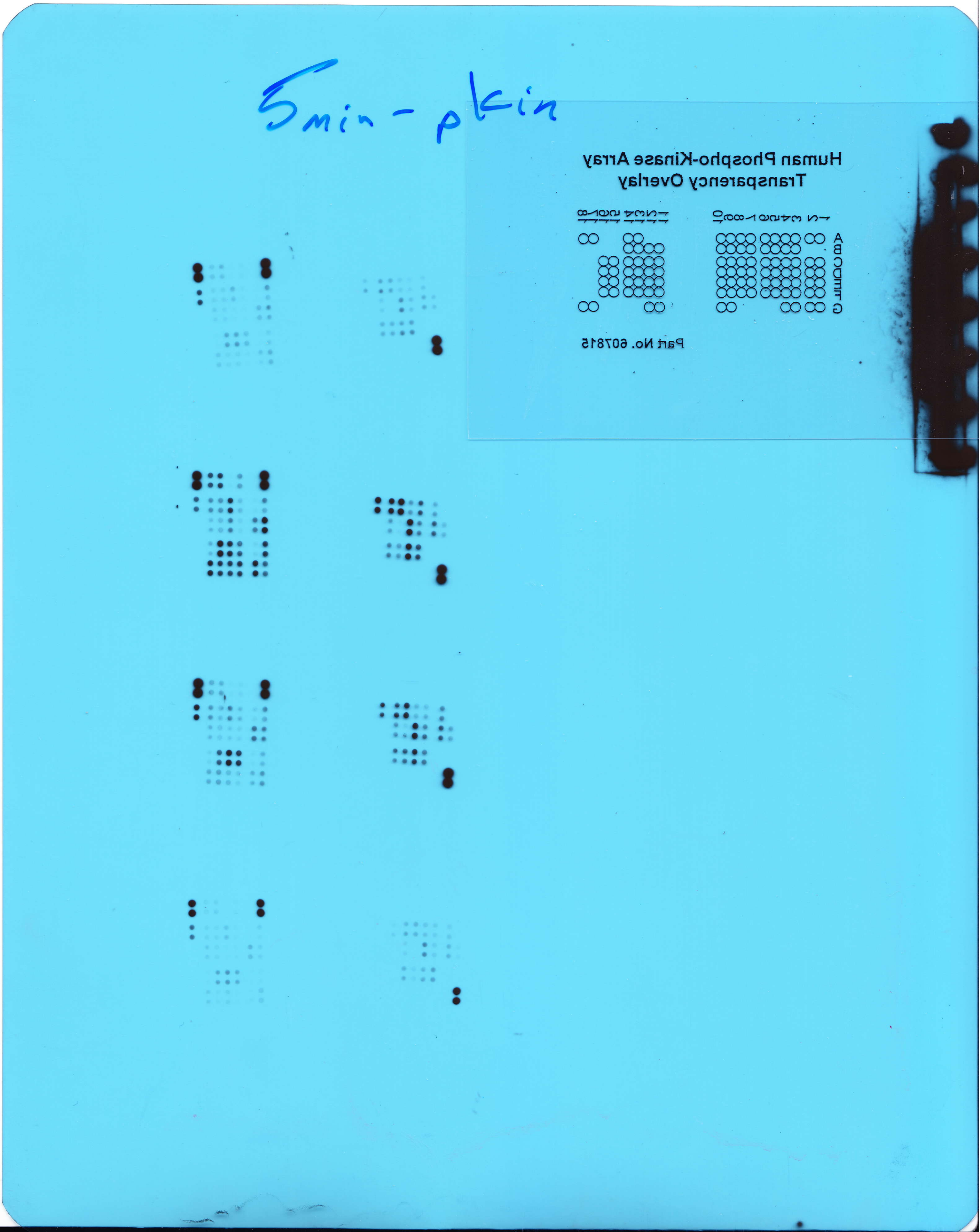
5min - pkin
Human Phospho-Kinase Array
Transparency Overlay
Part No. 607815

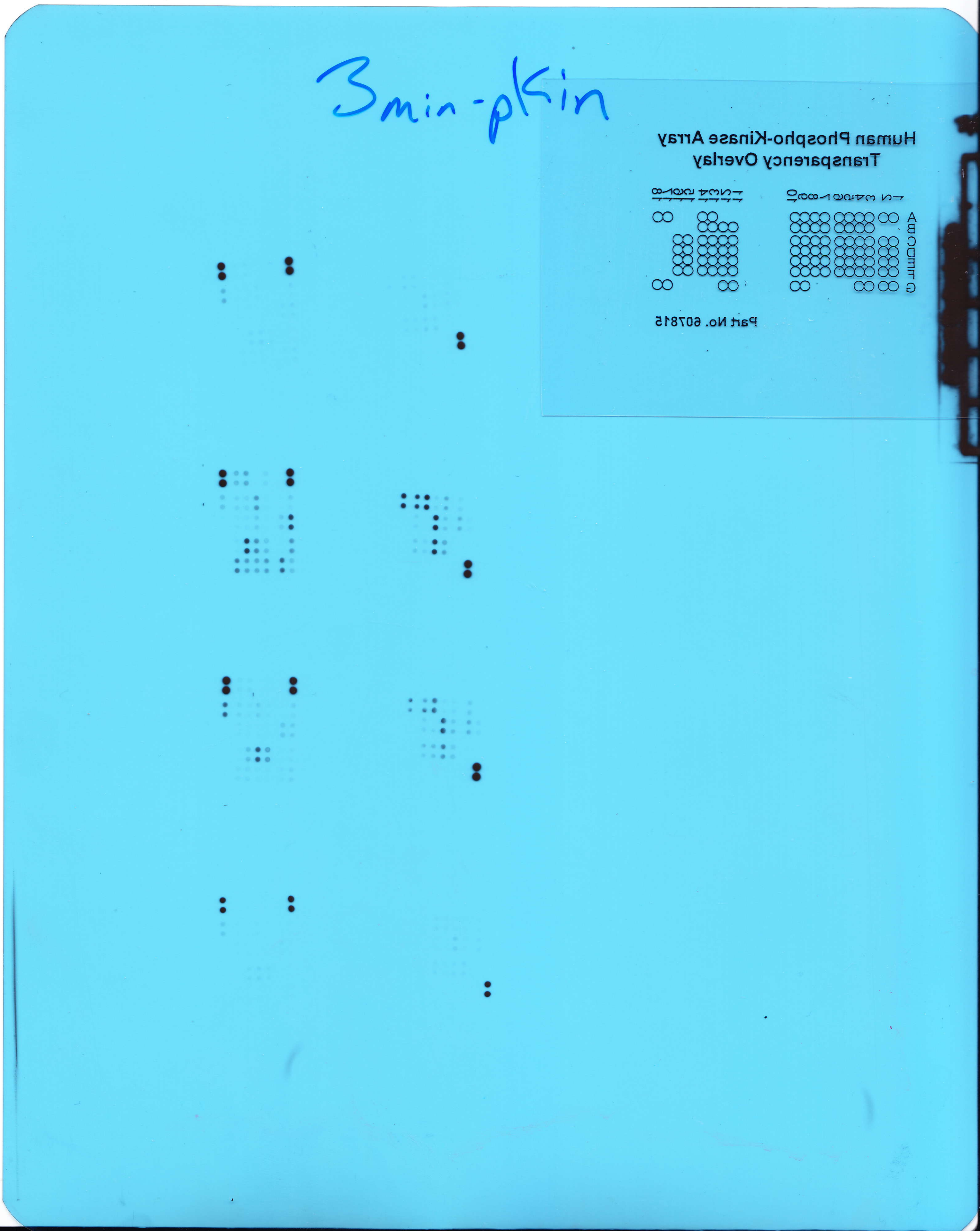
3min-pKin
Human Phospho-Kinase Array
Transparency Overlay
Part No. 607815

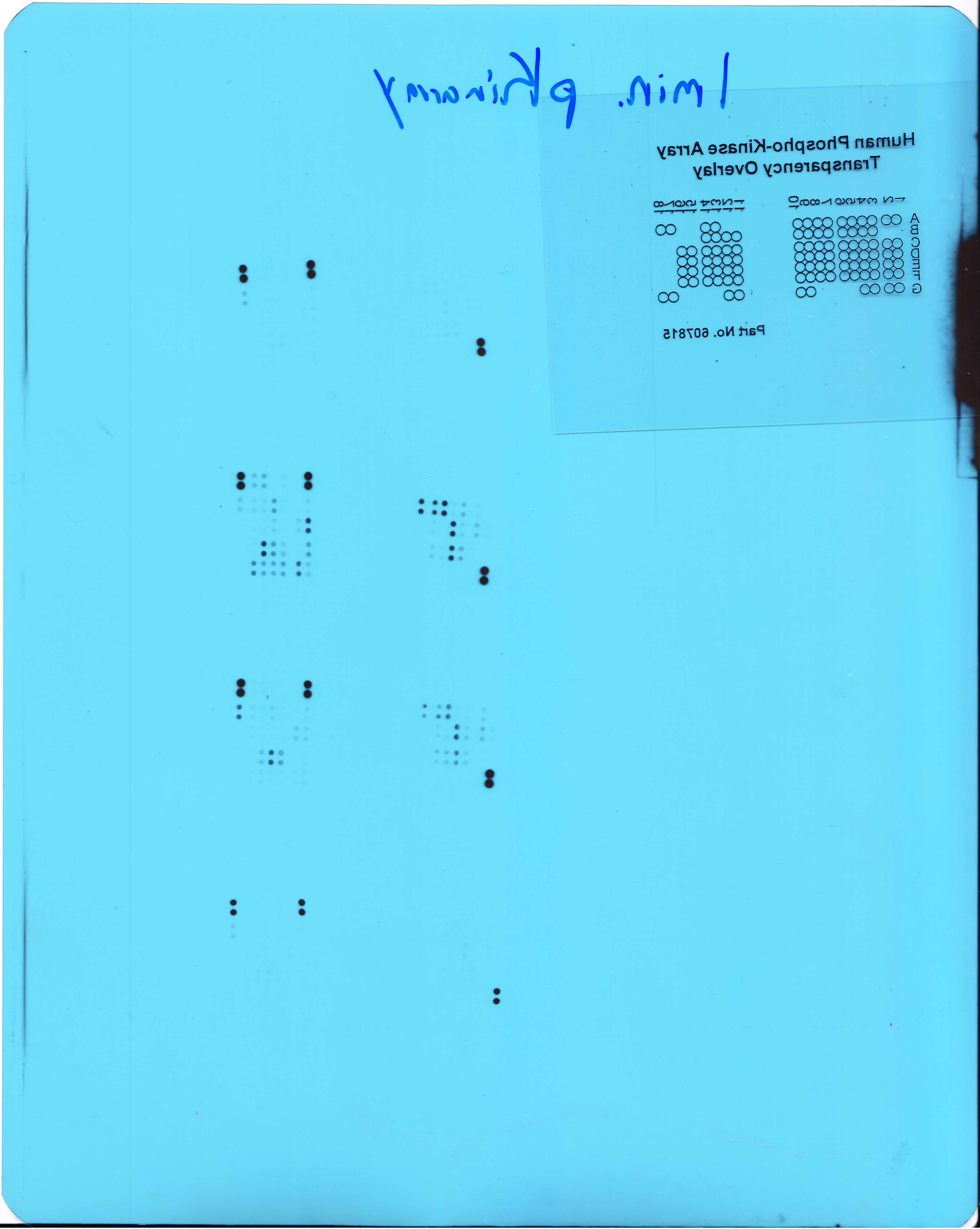
1min. primary
Human Phospho-Kinase Array
Transparency Overlay
A
B
C
D
E
F
G
Part No. 607815
