# Supplementary material for: Plasma Extracellular Vesicles Enriched for Neuronal Origin: A Potential Window into Brain Pathologic Processes
Source: Front Neurosci. 2017 May 22;11:278. doi: 10.3389/fnins.2017.00278 (PMC5439289; doi:10.3389/fnins.2017.00278)
Supplement: Supplemental Figure 3 — Original enhanced chemiluminescence (ECL) signal on film for Human MAPK Array (Figure 6C). [file Image3.PDF]

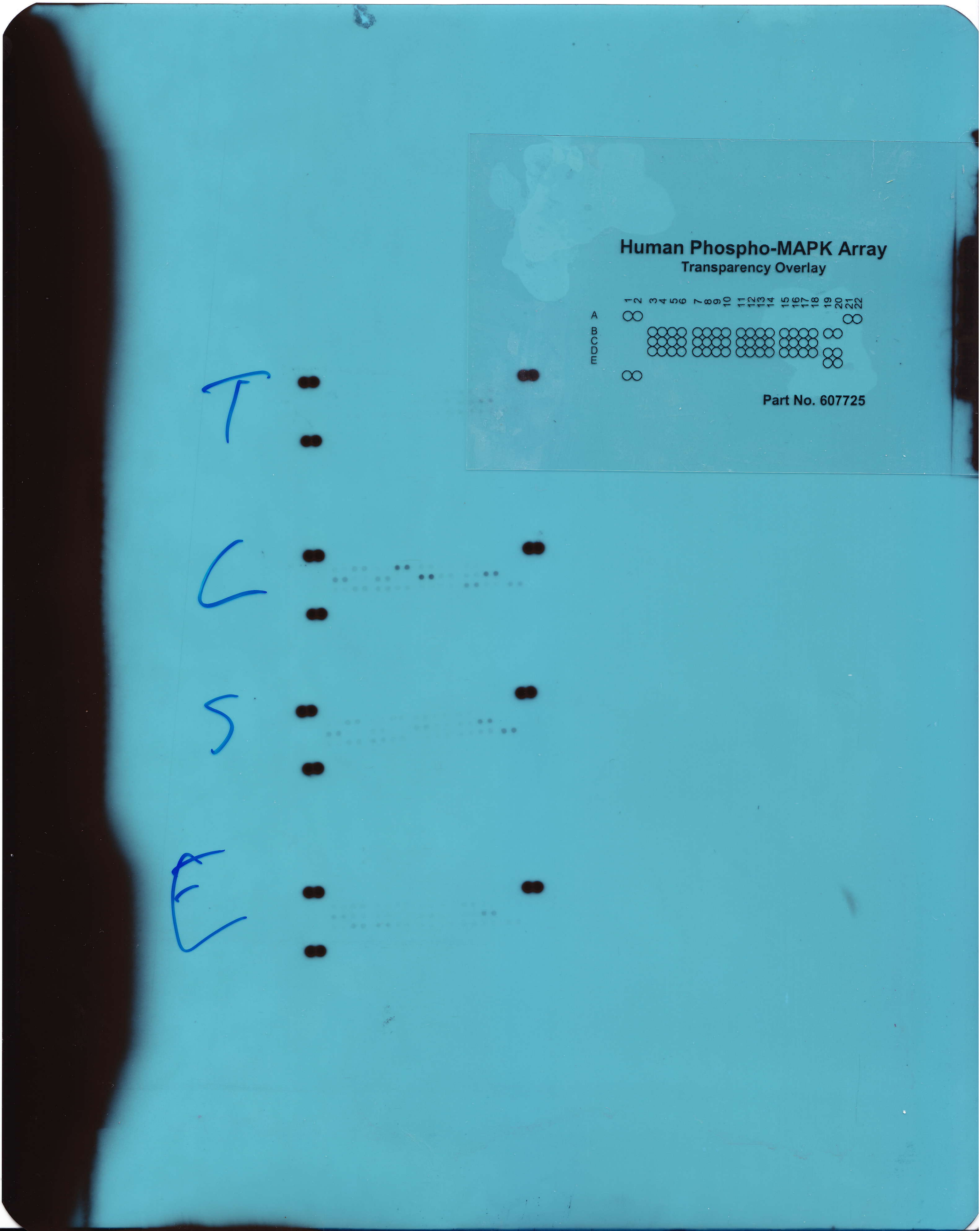
Human Phospho-MAPK Array
Transparency Overlay
A B C D E
1 2 3 4 5 6 7 8 9 10 11 12 13 14 15 16 17 18 19 20 21 22
Part No. 607725
T
C
S
E

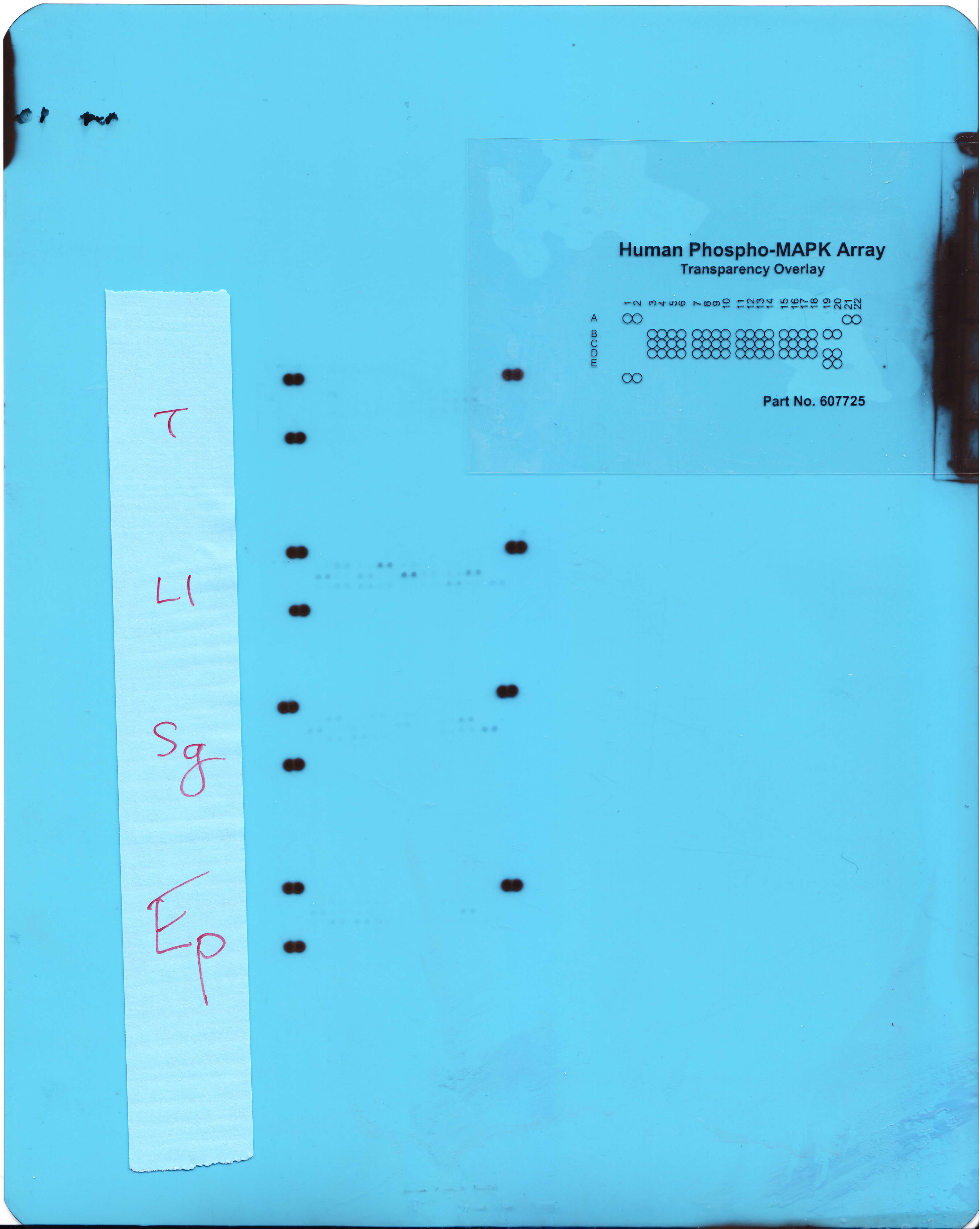
Human Phospho-MAPK Array
Transparency Overlay
Part No. 607725
T
L1
Sg
Ep
