# Supplementary material for: Plasma Extracellular Vesicles Enriched for Neuronal Origin: A Potential Window into Brain Pathologic Processes
Source: Front Neurosci. 2017 May 22;11:278. doi: 10.3389/fnins.2017.00278 (PMC5439289; doi:10.3389/fnins.2017.00278)

20min → Kidney Biomarker

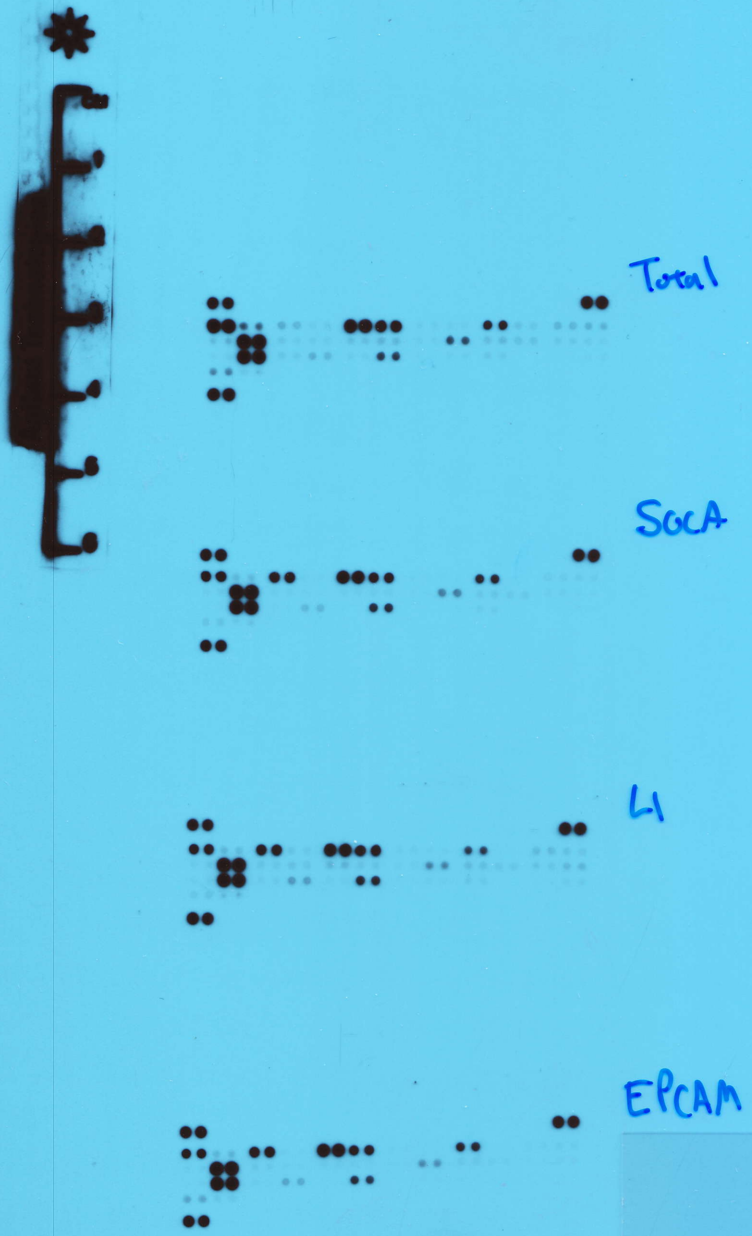

Human Kidney Biomarker Array
Transparency Overlay

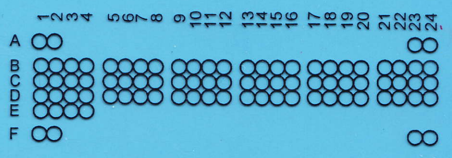

Part No. 607747

10 min

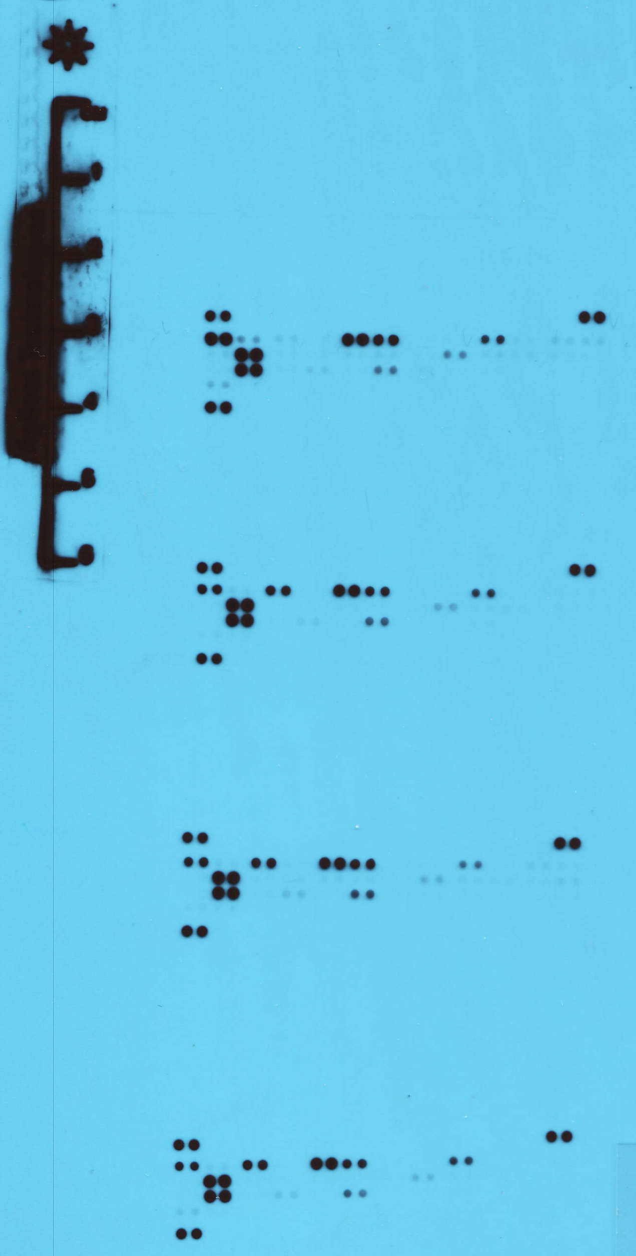

Human Kidney Biomarker Array
Transparency Overlay

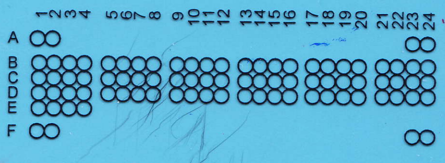

Part No. 607747

2 min Kidney Array

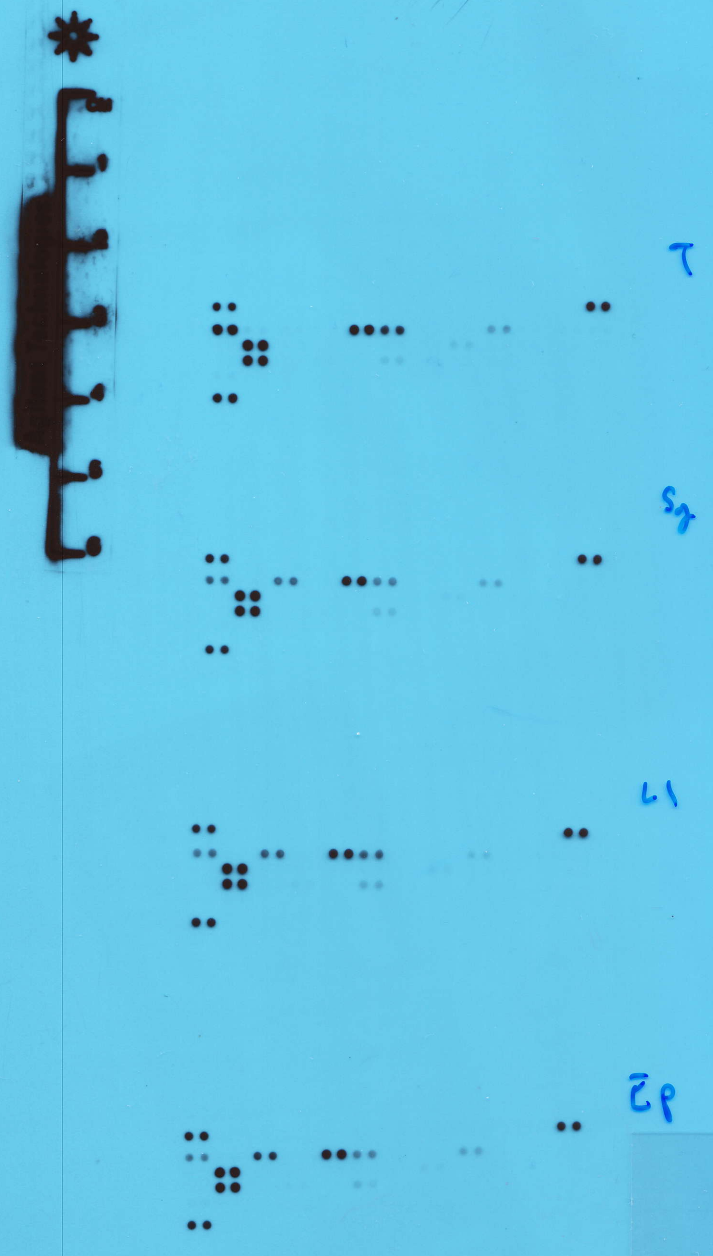

Human Kidney Biomarker Array Transparency Overlay

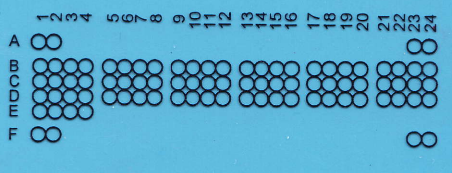

Part No. 607747

5min Kidney Array

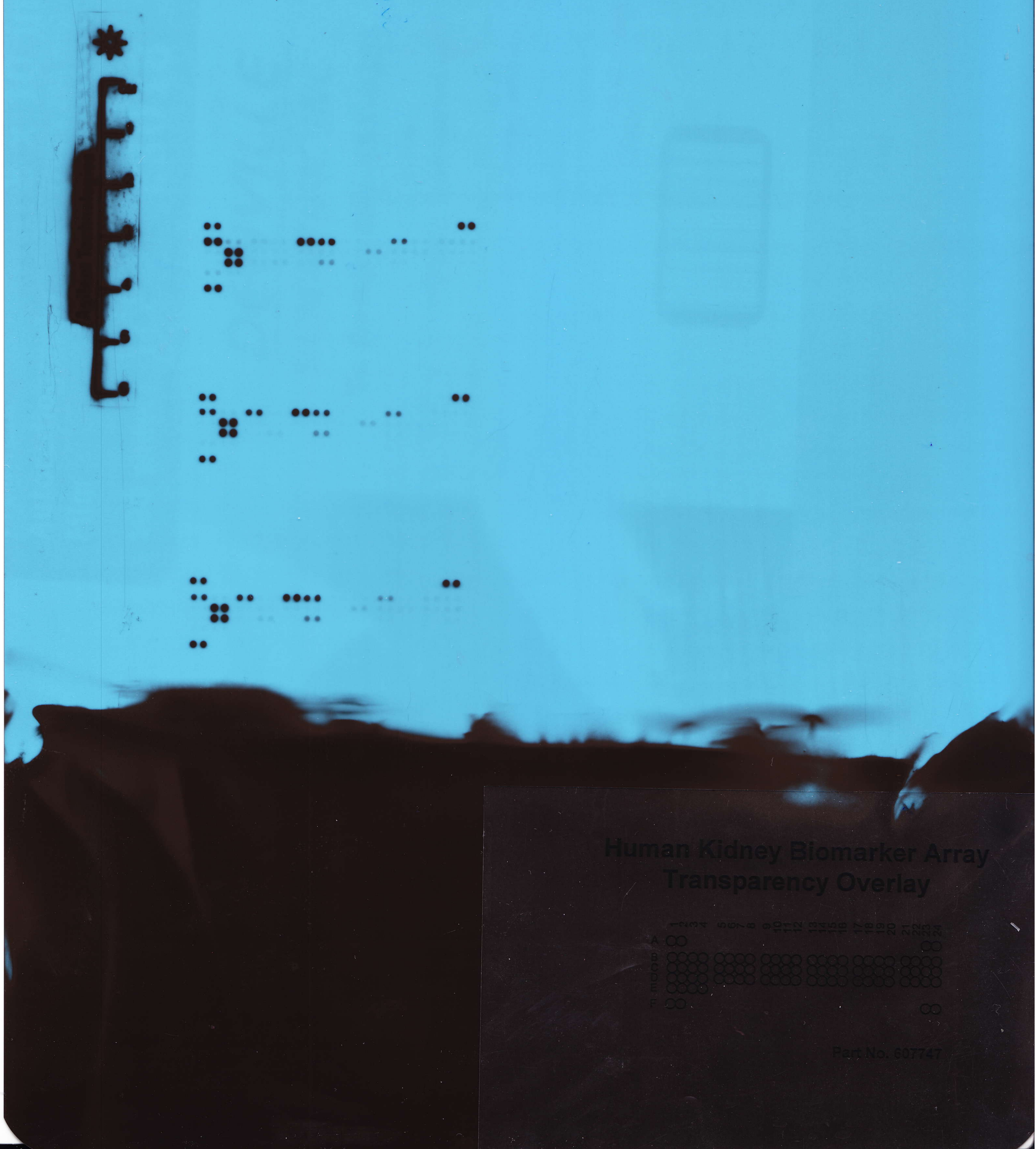

Supplement: Supplemental Figure 4 — Original enhanced chemiluminescence (ECL) signal on film for Human Kidney Biomarker Antibody Array (Figure 6D). [file Image4.PDF]
