# Supplementary material for: Plasma Extracellular Vesicles Enriched for Neuronal Origin: A Potential Window into Brain Pathologic Processes
Source: Front Neurosci. 2017 May 22;11:278. doi: 10.3389/fnins.2017.00278 (PMC5439289; doi:10.3389/fnins.2017.00278)
Supplement: Supplemental Figure 5 — Original enhanced chemiluminescence (ECL) signal on film for Human Apoptosis Antibody Array (Figure 6E). [file Image5.PDF]

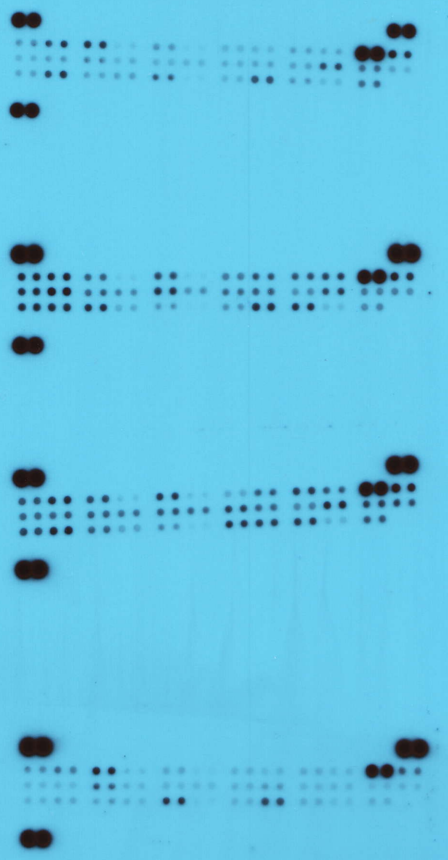

Human Apoptosis Array Transparency Overlay

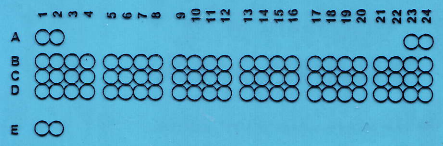

Part No. 607593

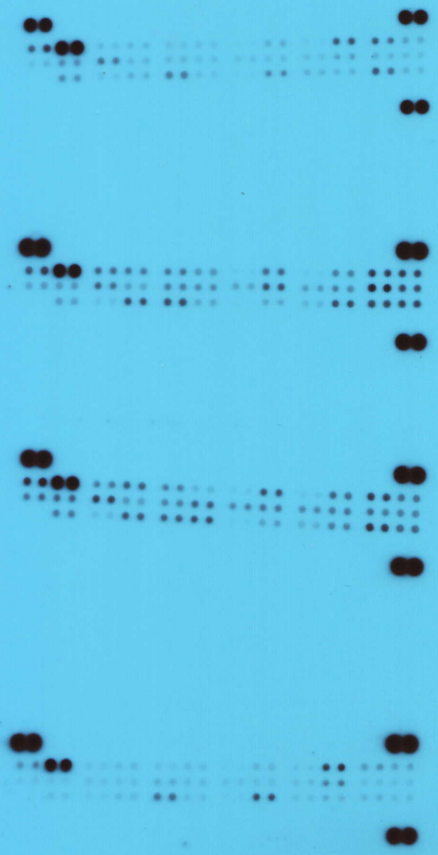

Human Apoptosis Array Transparency Overlay

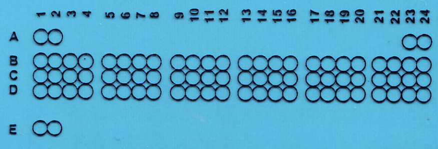

Part No. 607593

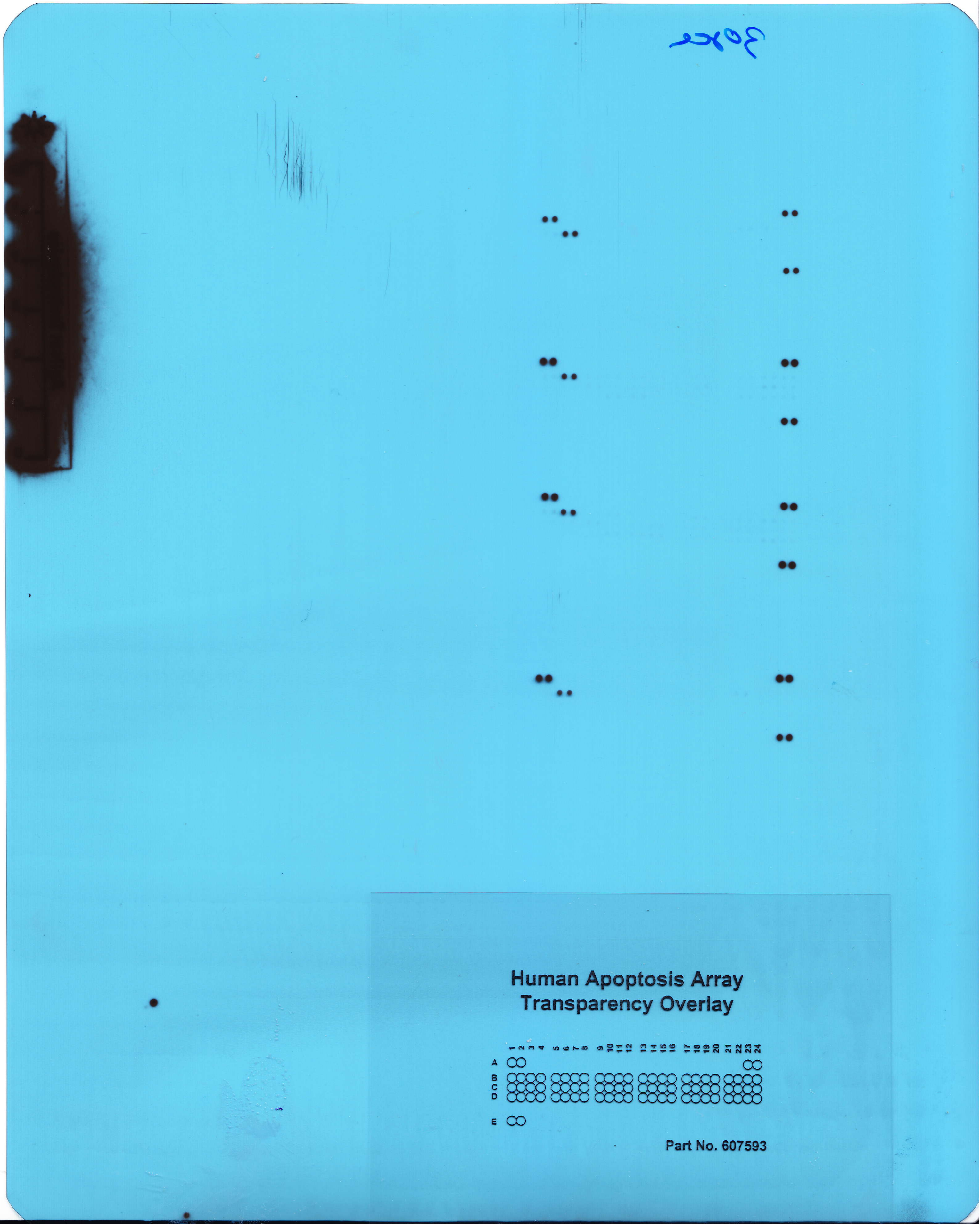
Human Apoptosis Array
Transparency Overlay
A
B
C
D
E
Part No. 607593

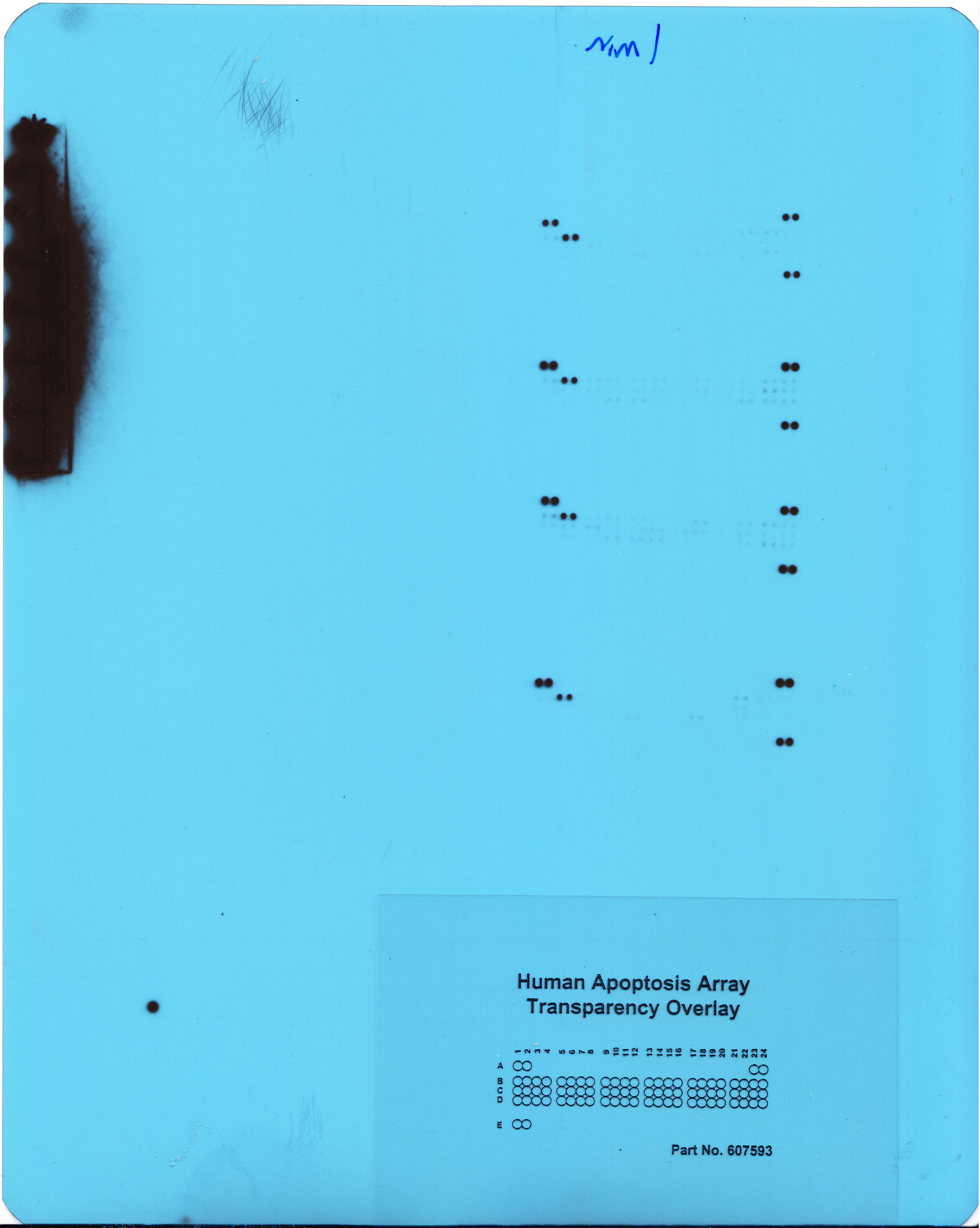
Human Apoptosis Array
Transparency Overlay
1 2 3 4 5 6 7 8 9 10 11 12 13 14 15 16 17 18 19 20 21 22 23 24
A
B
C
D
E
Part No. 607593
